# Molecular Docking of Natural Products: Critical Appraisal of Current Methodology and Practical Guidelines

**DOI:** 10.3390/molecules31183228

**Published:** 2026-09-12

**Authors:** Almagul S. Makhmutova, Nazigul S. Remetova, Gulnissa K. Kurmantayeva

**Affiliations:** School of Pharmacy, Karaganda Medical University, Karaganda 100024, Kazakhstan; remetnaziko@mail.ru (N.S.R.); gulnisa_s90@mail.ru (G.K.K.)

**Keywords:** molecular docking, natural products, molecular dynamics, docking validation, virtual screening, binding free energy, scoring functions, artificial intelligence, drug discovery, computational chemistry

## Abstract

Molecular docking is among the most widely used techniques in structure-based drug discovery and has become integral to natural-product research. Despite substantial advances in computational algorithms and artificial intelligence, the methodological quality of published docking studies on natural compounds remains highly variable, and generally accepted methodological guidelines have yet to be established. This review critically evaluates current approaches to molecular docking of natural compounds, examines the specific features of different natural-product classes and protein targets, and offers practical recommendations for the design and validation of docking studies. The review covers the main stages of molecular docking, contemporary search algorithms and scoring functions, protein and ligand preparation, the fundamental limitations of the method, strategies for result validation, and the emerging role of artificial intelligence in computational molecular modeling. A central component is a systematic methodological audit of publications within a prespecified 2025 coverage window, identified by Scopus and PubMed searches executed in August 2026. The audit assessed methodological reporting completeness only and was not intended as a systematic review of biological or pharmacological findings. The audit comprised GPT-assisted structured coding of 1127 publications, detailed full-text assessment of a random sample of 80 studies drawn entirely from the same corpus, and independent human validation of 20 randomly selected Stage 2 publications. In the 80-publication full-text sample, redocking was reported in 7.5% of publications, a qualifying redocking RMSD below 2 Å in 6.2%, positive controls in 87.5%, molecular dynamics in 41.2%, MM/PBSA or MM/GBSA in 12.5%, interaction analysis in 98.8%, and ADMET assessment in 35.0%. Agreement between Stage 1 and Stage 2 was 98.3% across 525 paired criterion decisions. Independent human assessment of the 20-publication validation subsample agreed with Stage 2 in 135 of 140 criterion decisions (96.4%); the five discrepancies all involved AI-coded indeterminate/non-confirmed labels (Unconfirmed or N/A) that the human reviewer classified as No. On the basis of these findings, we propose a practical workflow aimed at improving the reproducibility and methodological rigor of molecular docking studies of natural compounds. A complementary targeted case-enriched validation of redocking and redocking-RMSD classification included 19 publications and two human reviewers. The reviewers reached identical classifications in all 38 criterion decisions; their consensus agreed with Stage 2 in 31 of 38 decisions (81.6%), with seven revisions across four publications. Because this sample was deliberately enriched for informative and ambiguous cases, it was used to examine classification boundaries rather than to estimate prevalence. Because PubMed retrieval was restricted to free full text and the Scopus search used restricted bibliographic fields, these reporting-completeness and validation frequencies primarily characterize an accessibility-enriched corpus and may not fully generalize to subscription-only or otherwise less-accessible journals. By combining a critical appraisal of current methods with a quantitative assessment of published studies and practical recommendations for standardizing the molecular docking of natural compounds, this review offers guidance for researchers in computer-aided drug design.

## 1. Introduction

Natural compounds have been an important source of drug leads throughout the history of pharmacology. Despite the rapid development of combinatorial chemistry, high-throughput synthesis, and rational drug-design strategies, a substantial proportion of small-molecule therapeutics approved for clinical use are still derived, either directly or indirectly, from natural sources. According to the widely cited analysis by Newman and Cragg covering the period from 1981 to 2019, natural products, their semisynthetic derivatives, and synthetic mimetics continue to make major contributions to drug discovery, particularly in oncology and antibacterial and antifungal therapy [1]. The pronounced structural complexity of natural metabolites, shaped by biological evolution, enables a broad range of spatial and electronic interactions with protein targets, thereby expanding the chemical space available for identifying novel pharmacologically active compounds [2,3].

### 1.1. Role of Computational Methods in Natural-Product Discovery

Conventional discovery of biologically active natural substances remains a lengthy and costly process involving compound isolation, structural elucidation, and subsequent experimental testing. At the same time, the volume of information available on natural metabolites continues to increase owing to advances in mass spectrometry, metabolomics, biosynthetic gene-cluster genomics, and specialized chemical databases [2,3]. Computer-aided drug-design methods have consequently become integral to contemporary discovery strategies, reducing the number of candidates requiring experimental evaluation and allowing research efforts to focus on the most promising molecules [4].

### 1.2. Molecular Docking as a Central Tool in Virtual Screening

Among structure-based drug-design methods, molecular docking is a central tool because it can predict the three-dimensional arrangement of a protein-ligand complex and estimate potential binding affinity before laboratory experiments are performed [4,5]. Unlike ligand-based approaches, docking does not require prior information on a series of known active compounds and can therefore be applied to poorly characterized protein targets and new classes of natural compounds. It is now widely used in virtual screening, drug repurposing, the investigation of multitarget activity and protein–protein interactions, and the identification of potential molecular targets [5].

Molecular docking is particularly valuable in studies of natural compounds, which exhibit pronounced structural diversity, numerous stereogenic centers, and complex three-dimensional architectures. However, these same characteristics considerably complicate ligand preparation, selection of an appropriate computational protocol, and interpretation of the resulting binding modes, thereby necessitating dedicated methodological approaches [6].

### 1.3. Current Methodological Challenges in Molecular Docking Studies of Natural Compounds

Despite the rapid growth in the number of publications addressing the molecular docking of natural compounds, the methodological quality of these studies remains highly heterogeneous. Reports lacking redocking, quantitative validation of the computational protocol, appropriate positive controls, or assessment of complex stability are still common. Concurrently, advances in artificial intelligence and the emergence of AlphaFold, DiffDock, neural-network-based scoring functions, and new computational platforms have substantially expanded the capabilities of molecular modeling while also increasing the need for rigorous evaluation of prediction reliability.

Most published reviews focus either on molecular docking algorithms, individual classes of natural compounds, or specific diseases. By contrast, very few studies integrate an analysis of contemporary computational approaches with a systematic assessment of the methodological quality of published docking investigations involving natural compounds. This gap substantially limits reproducibility and hampers the development of consistent recommendations for conducting such studies.

## 2. Evolution of Molecular Docking

Over the past four decades, molecular docking has evolved from simple algorithms designed to identify geometric complementarity between rigid molecules into sophisticated hybrid platforms integrating physics-based models, empirical scoring functions, and artificial-intelligence methods [7,8,9,10,11,12,13,14].

Contemporary docking programs can account for ligand conformational flexibility and, to a limited extent, receptor flexibility; perform large-scale virtual screening of millions of compounds; and apply machine-learning methods to improve the accuracy of complex ranking [8,9,10,11,12]. The field has also been profoundly influenced by AlphaFold, which has enabled docking studies of proteins lacking experimentally determined structures [13], and by a new generation of generative models such as DiffDock [14].

Nevertheless, despite substantial advances in computational algorithms, the principal limitations of molecular docking remain the appropriate preparation of proteins and ligands, selection of a suitable scoring function, and the lack of comprehensive experimental validation. These issues are discussed in detail in the following sections.

## 3. Principal Stages of Molecular Docking

Molecular docking is not a single computational operation but a sequence of interdependent steps, each contributing to the overall prediction error. Errors introduced at an early stage, such as target selection or receptor preparation, generally cannot be compensated for by even the most accurate sampling algorithm or scoring function at a later stage. The key steps involved in docking natural compounds are discussed below.

### 3.1. Protein-Target Selection

The reliability of a molecular docking study depends largely on the quality of the selected three-dimensional protein structure. Errors introduced at this stage cannot be fully compensated for by subsequently applying more sophisticated pose-search algorithms or scoring functions. Receptor selection is thus a critical step in any computational investigation.

#### 3.1.1. The Protein Data Bank as the Primary Source of Structures

The Protein Data Bank (PDB), the world’s largest repository of three-dimensional structures of biological macromolecules, remains the primary source of experimentally determined protein structures [15]. In addition to atomic coordinates, a PDB entry provides information on the structure-determination method, resolution, composition of the complex, and the presence of ligands, cofactors, metal ions, and mutations. The suitability of an individual entry for molecular docking is determined by this complete set of characteristics rather than merely by its availability in the database.

#### 3.1.2. AlphaFold and Predicted Structures

The introduction of AlphaFold2 substantially expanded the range of proteins available for structure-based modeling, while the AlphaFold Protein Structure Database made millions of predicted structures accessible, including those of proteins with no experimentally determined counterparts [13,16]. This development is particularly relevant to natural-product research, in which potential targets frequently belong to poorly characterized protein families.

However, AlphaFold models do not overcome the fundamental limitations of molecular docking [17]. High accuracy of the overall protein fold does not guarantee correct representation of the local geometry of the active site, particularly in regions containing flexible loops or amino-acid side chains. Accordingly, when an experimental structure is available, it should generally be preferred; AlphaFold models are most appropriately used for proteins that are not represented in the PDB.

#### 3.1.3. Criteria for Structure Selection

Several factors should be considered when selecting a PDB entry. High-resolution structures are generally preferable because the accuracy with which amino-acid side chains are positioned directly affects the modeling of protein-ligand interactions [18]. In addition to resolution, statistical indicators of structure quality (R and Rfree), alternative conformations, model completeness, and the presence of a co-crystallized ligand should be evaluated.

Missing protein regions, engineered mutations, and features of the experimental construct should also be taken into account. When such alterations involve the active site or adjacent regions, they may substantially modify binding-pocket geometry and introduce systematic docking errors. In these cases, an alternative structure should preferably be selected or the missing segments modeled before docking.

Protein-structure selection is not merely a routine preparatory task; it is a major determinant of the reliability of subsequent calculations. The quality of the initial receptor model should be evaluated as carefully as the molecular docking results themselves.

### 3.2. Protein Preparation

Even a high-quality experimental structure cannot be used for molecular docking without prior preparation. Crystallographic models contain features arising from experimental conditions that may substantially affect modeling outcomes. Available evidence indicates that errors introduced during receptor preparation can have a greater effect on virtual-screening performance than the choice of docking algorithm or scoring function [19].

#### 3.2.1. Receptor-Structure Preparation

Protein preparation typically includes removing irrelevant water molecules, adding hydrogen atoms, assigning protonation states and partial charges, and verifying the treatment of cofactors and metal ions. Solvent removal should not, however, be fully automated: conserved water molecules involved in active-site hydrogen-bonding networks may contribute substantially to ligand binding and should be retained when appropriate [19].

Particular attention should be paid to the protonation states of amino-acid residues. Incorrect assignment of charges to catalytic residues or metal-coordinating histidines can substantially alter intermolecular interactions and lead to erroneous compound ranking.

#### 3.2.2. Cofactors and Metalloproteins

When enzymes containing cofactors or metal ions are investigated, these components should be treated as integral parts of the receptor. Removal of haem groups, NAD(P), FAD, catalytic Zn^2+^ or Mg^2+^ ions, or other metals may distort active-site geometry and reduce the reliability of the results. Because conventional scoring functions do not always describe metal-ligand coordination accurately, docking results for metalloproteins require particularly cautious interpretation.

#### 3.2.3. Definition of the Docking Region

Selection of the search region (grid box) is equally important. The most reliable approach is to define it using the position of a co-crystallized ligand or known catalytic residues. When experimental information is unavailable, potential binding pockets can be identified using computational methods such as fpocket, which analyses protein-surface geometry [20].

Incorrect identification of the active site renders subsequent calculations meaningless, irrespective of the docking software used [21]. Defining the search region is therefore one of the most consequential steps in receptor preparation.

#### 3.2.4. Summary

Appropriate protein preparation is essential for obtaining reproducible molecular docking results. Many methodological errors arise not from the docking algorithm itself but from inadequate receptor preparation; the quality of this step should be assessed as rigorously as the results of the subsequent modeling.

### 3.3. Preparation of Natural Compounds

Ligand preparation is one of the most critical stages of molecular docking because it determines the correctness of the molecular geometry, ionization state, and conformational space explored. In contrast to many synthetic drug-like molecules, natural metabolites frequently exhibit pronounced stereochemical complexity, numerous rotatable bonds, and several plausible tautomeric or protonation states, necessitating more rigorous preprocessing.

#### 3.3.1. Natural-Compound Databases

PubChem [22], ChEMBL [23], ZINC [24], NPASS [25], and COCONUT [26] are among the databases most frequently used as sources of natural-compound structures. These resources differ in scope and functionality: PubChem provides the broadest coverage of chemical structures; ChEMBL contains experimental bioactivity data; ZINC offers libraries prepared for virtual screening; and NPASS and COCONUT focus primarily on natural compounds and facilitate the construction of targeted docking libraries.

#### 3.3.2. Protonation and Tautomerism

Correct assignment of the protonation state and tautomeric form is an essential component for ligand preparation. This is particularly important for alkaloids, flavonoids, and other natural compounds containing ionizable functional groups. Programs such as Epik can automatically generate the most probable protonation states within a specified pH range [27]. Restricting the analysis to a single arbitrarily selected form can introduce substantial errors into binding-affinity estimates.

#### 3.3.3. Generation of Three-Dimensional Structures and Conformers

After converting a two-dimensional representation into a three-dimensional structure, geometry optimization and generation of an ensemble of low-energy conformers should be performed. Specialized algorithms such as OMEGA are widely used for this purpose because they provide representative coverage of conformational space and increase the likelihood of identifying a biologically relevant binding pose [28]. This step is particularly important for flexible natural compounds, including saponins, polyphenols, and macrocyclic structures.

#### 3.3.4. Stereochemistry

The stereochemistry of natural compounds requires separate verification. Many secondary metabolites contain several stereogenic centers, and their biological activity depends on a specific absolute configuration. Use of a structure with incorrectly assigned stereochemistry, or automated generation of undefined stereocenters, may result in docking a compound that does not actually occur in nature. Consistency between the modeled structure and the stereochemical information reported in the literature should be considered an essential component of ligand preparation.

### 3.4. Molecular Docking Software

Selection of a docking program requires balancing computational speed, pose-prediction and/or affinity-ranking accuracy, availability (open-source or commercial), and compatibility with downstream analyses. Table 1 compares nine programs that are widely used in the literature on molecular docking of natural compounds. Because available benchmarks differ substantially in datasets and endpoints, Table 1 summarizes algorithms, practical advantages, and limitations without imposing a universal speed or accuracy ranking.

Speed and pose-prediction accuracy are not assigned universal qualitative categories in Table 1. Their relative performance depends on the benchmark design, target class, ligand set, search settings, hardware, and evaluation metric; direct labels such as “high” or “very high” can be misleading, particularly when classical programs are compared with newer machine-learning approaches such as GNINA and DiffDock.

A general trade-off between docking speed and accuracy is evident across the field. Deep-learning approaches such as GNINA and DiffDock overcome this trade-off only partly, because their practical accuracy depends strongly on the similarity of the target and ligand class to the training data. Natural compounds are often structurally atypical relative to the synthetic drug-like molecules that predominate in the PDB and in training datasets. Results obtained using neural-network-based methods should be interpreted with particular caution, as discussed further in the critical appraisal of methodology.

## 4. Scoring Functions in Molecular Docking

A scoring function is a key component of any molecular docking program because it ranks the generated ligand poses and estimates their putative binding affinity. Despite major advances in computational algorithms, no universal scoring function performs equally well across all proteins and classes of natural compounds. Current approaches can be broadly divided into four groups: force-field-based, empirical, knowledge-based, and artificial-intelligence-based functions.

### 4.1. Force-Field-Based Scoring Functions

Force-field-based scoring functions are grounded in classical molecular mechanics and describe protein-ligand interactions as the sum of van der Waals and electrostatic terms and, in some cases, ligand strain energy. This approach was implemented in the earliest generations of molecular docking software, including early versions of DOCK and AutoDock [7,10].

Their main advantage is physical interpretability and the ability to use parameters from well-established force fields such as AMBER, CHARMM, and MMFF. They are therefore relatively transferable to new chemical classes and do not depend directly on the composition of training datasets.

However, these functions provide only approximate descriptions of desolvation, entropy, and cooperative interactions. Moreover, additive models do not adequately reproduce complex intermolecular contacts, including hydrogen-bond networks, pi-pi stacking, and cation-pi interactions, which are common in complexes involving natural compounds. Force-field-based functions therefore generally provide satisfactory ligand-pose predictions but are considerably less reliable for quantitative estimation of binding energy.

### 4.2. Empirical Scoring Functions

Empirical scoring functions represent binding energy as a weighted sum of individual interaction terms, including hydrogen bonds, ionic contacts, hydrophobic interactions, and entropic corrections. The corresponding coefficients are obtained by statistical fitting to experimental protein-ligand complexes with known affinities. Classic examples include the Bohm function implemented in LUDI [30] and ChemScore, which is widely used in GOLD [31].

The main advantages of empirical functions are their computational efficiency and relatively good correlation with experimental measurements for compounds chemically similar to those in the training set. Individual terms also retain a physical meaning, facilitating interpretation and subsequent structural optimization of potential ligands.

Nevertheless, their accuracy depends strongly on training-set composition. Because most empirical functions were developed primarily using synthetic drug-like molecules, they may describe structurally complex natural metabolites containing polyphenolic moieties, macrocycles, glycosides, and other atypical features less accurately. A linear additive model also poorly captures nonlinear and cooperative binding effects, limiting the general applicability of these functions.

### 4.3. Knowledge-Based Scoring Functions

Knowledge-based scoring functions are derived from large collections of experimentally determined protein-ligand complexes deposited in the Protein Data Bank. Unlike force-field-based and empirical models, they do not calculate interaction energies directly; instead, they use observed frequencies of interatomic contacts to derive potentials of mean force (PMFs). A prominent example is the Muegge-Martin PMF, implemented, among other programs, in DOCK [32].

A major advantage of knowledge-based functions is their implicit representation of solvation and entropic effects encoded in experimental structural statistics. These models are computationally efficient and can perform well in virtual-screening applications.

Their accuracy, however, depends directly on the composition of the structural database. Because the PDB is dominated by complexes containing synthetic drug-like molecules, statistical potentials may inadequately represent structural motifs that are uncommon but characteristic of natural metabolites, such as unusual heterocycles, glycosidic residues, and macrocyclic scaffolds. Performance also depends on the choice of reference state, which remains a major point of debate in the development of statistical scoring functions [32].

### 4.4. AI-Based Scoring Functions

Advances in machine learning have produced a new generation of scoring functions capable of identifying complex nonlinear relationships between complex structure and binding energy. Unlike conventional models, AI algorithms do not assume a predefined functional form but learn directly from large experimental datasets. RF-Score, based on a random-forest algorithm, was among the first successful examples [33], whereas contemporary programs such as GNINA use ensembles of convolutional neural networks to rescore docked poses [12].

AI-based scoring generally provides more accurate complex ranking on standard benchmark datasets than most conventional functions. Its performance nevertheless depends strongly on the quality and representativeness of the training data. Most current algorithms were trained predominantly on complexes containing synthetic drug-like molecules, whereas structurally complex natural compounds are markedly underrepresented. This limits extrapolation to new chemical classes and necessitates cautious interpretation of computational estimates.

Limited interpretability is an additional concern. Despite high predictive accuracy, neural-network models operate largely as black boxes, complicating assessment of the contributions of individual interactions and rational optimization of potential leads. Models may also overfit idiosyncrasies of the training data; consequently, high accuracy on standard benchmarks is not invariably reproduced for new protein targets or natural compounds [18].

### 4.5. Summary

The scoring-function classes considered above differ substantially in their computational foundations, yet none provides universally accurate binding-affinity predictions for all proteins and natural-product classes. Force-field-based functions are physically interpretable but describe solvation and entropy only approximately. Empirical models are computationally efficient, but their accuracy depends on representative training data. Knowledge-based functions exploit experimental structural information effectively but are sensitive to PDB composition, whereas AI-based models provide the highest ranking accuracy chiefly within the chemical space represented in their training datasets.

These limitations are particularly important for natural compounds, which frequently exhibit pronounced structural complexity and differ substantially from the synthetic molecules used to develop most current scoring functions. Calculated docking scores should therefore be interpreted primarily as comparative ranking metrics rather than direct quantitative measures of biological activity.

Selection of a scoring function should consequently reflect the characteristics of the protein target, the chemical class of the natural compounds, and the objective of the study. Combined approaches integrating molecular docking with molecular dynamics, binding free-energy calculations, and experimental validation are increasingly used and are discussed in Section 7 and Section 9.

## 5. Molecular Docking of Different Classes of Natural Compounds

The diversity of natural compounds is a major advantage in the search for new drug candidates but also poses a substantial computational challenge. Differences in molecular mass, three-dimensional organization, number of stereogenic centers, protonation state, and conformational mobility affect every stage of a docking study, from ligand preparation (Section 3.3) to interpretation of binding energies and subsequent validation (Section 7 and Section 9). A single universal docking protocol is thus inappropriate for all natural-product classes.

### 5.1. Alkaloids

Alkaloids are among the most extensively studied natural-product classes because of their pronounced biological activity and ability to interact with a broad range of molecular targets [1,34]. However, the presence of one or more nitrogen atoms makes this class particularly susceptible to ligand-preparation errors.

Incorrect assignment of protonation states is the most frequent source of erroneous results. At physiological pH, many tertiary amines exist predominantly in a protonated form, yet published studies often model them as neutral species. This substantially alters electrostatic interactions and calculated binding energies [27]. Alkaloid preparation must therefore include prediction of the most probable ionization states, as discussed in Section 3.3.

Stereochemistry is a second critical factor. Many alkaloids contain several stereogenic centers, and use of an incorrect absolute configuration can completely alter the predicted binding pose. Verification of stereochemistry and subsequent validation of the resulting complexes by molecular dynamics or binding-free-energy calculations are therefore particularly important for this class (Section 9) [27].

### 5.2. Flavonoids

Flavonoids are the most widely represented class of natural compounds in molecular docking studies [35]. Despite their relatively low molecular mass, they may display substantial conformational variability and contain numerous hydroxyl groups, both of which materially affect computational modeling.

Accurate description of conformational space is a central requirement in flavonoid docking. Rotation of the pendant aromatic ring and the presence of multiple hydrogen-bond donors and acceptors necessitate generation of an ensemble of low-energy conformers before docking (Section 3.3) [28].

Glycosylated derivatives present particular difficulties. Sugar residues markedly increase the number of rotatable bonds and reduce the efficiency of conventional docking algorithms. Classical scoring functions may also overestimate the contribution of numerous hydrogen bonds, as discussed among the limitations of the method (Section 7) [30,31,32]. Flavonoid docking results should preferably be supported by molecular dynamics and MM/GBSA or MM/PBSA calculations (Section 9).

### 5.3. Terpenoids

Terpenoids are characterized by highly three-dimensional structures, numerous stereogenic centers, and diverse polycyclic scaffolds [1,36]. These properties promote spatial complementarity with hydrophobic protein regions but also complicate ligand preparation.

Generation of correct three-dimensional geometry is the most serious computational challenge. Automated preparation algorithms do not always reproduce bridged and fused ring systems accurately, allowing errors to arise before docking itself [28]. Terpenoid conformations should therefore undergo visual inspection, and several independent docking runs should be performed.

Interpretation of binding energy is another source of uncertainty. Complex stabilization for many terpenoids is dominated by hydrophobic interactions, whereas hydrogen bonds often make a secondary contribution. Complex quality should be evaluated not only from the docking energy but also from the interaction pattern and its persistence during molecular dynamics simulations (Section 7 and Section 9) [36].

### 5.4. Xanthones

Xanthones are particularly suitable for molecular docking because of their relatively rigid polycyclic structure and restricted conformational mobility [37]. Compared with more flexible natural-product classes, they require less extensive conformational sampling and generally yield more reproducible predicted binding poses.

Xanthone-protein complexes are stabilized primarily by pi-pi interactions with aromatic amino acid residues and by hydrogen bonds formed by hydroxyl substituents. Correct assignment of protonation states and tautomeric forms remains essential (Section 3.3) [27].

Prenylated derivatives present the greatest computational challenge because their flexible side chains substantially expand conformational space. Extensive conformer generation and evaluation of several probable binding poses, rather than reliance on a single docking solution, are recommended in such cases [28].

Overall, xanthones are well suited to the assumptions of conventional molecular docking and can serve as a model class for evaluating protocol performance.

### 5.5. Coumarins

Coumarins are widely used in molecular docking studies because of their small size, restricted conformational mobility, and favorable drug-like properties [38].

Despite their structural simplicity, docking results require cautious interpretation. The activity of some coumarins is determined not only by noncovalent binding but also by potential chemical modification of the protein or hydrolysis of the lactone ring. Because conventional programs model only noncovalent interactions, calculated binding energies may not reflect the actual mechanism of action [38].

The docking score should therefore be considered together with the ligand position relative to catalytic residues; when experimental evidence is available, alternative mechanisms of inhibition should also be considered (Section 7).

### 5.6. Polyphenols

Polyphenols constitute one of the largest groups of natural compounds used in virtual screening because they can form multiple hydrogen bonds with proteins [35,39].

This class is, however, particularly sensitive to the limitations of contemporary scoring functions. Numerous hydroxyl groups can produce systematic overestimation of binding energy because most algorithms do not adequately account for the desolvation penalty associated with polar functional groups (Section 7) [30,31,32].

The high molecular mass of some members, particularly condensed tannins and other oligomeric polyphenols, creates an additional problem. Conventional docking algorithms, developed primarily for relatively small drug-like molecules, perform less effectively for such compounds [18].

Polyphenol docking is best regarded as an initial ranking step. Molecular dynamics and binding-free-energy calculations are recommended for the most promising complexes to reduce the impact of limitations inherent in classical scoring functions (Section 9) [39].

### 5.7. Lignans

Lignans are a structurally diverse group of phenylpropanoid dimers widely investigated as potential anticancer, anti-inflammatory, and antioxidant agents [40]. From a docking perspective, they occupy an intermediate position between relatively rigid aromatic compounds and highly flexible natural ligands.

A defining feature of lignans is the strong conformational dependence of docking results. Cyclized members are generally described adequately by conventional algorithms, whereas open dibenzylbutane structures possess substantially greater rotational freedom and require extensive conformer generation during ligand preparation (Section 3.3) [28].

The presence of two aromatic moieties also increases the likelihood of several energetically similar binding modes. For lignans, both the top-ranked pose and alternative complexes with closely related energies should therefore be examined, followed by molecular-dynamics assessment of their stability (Section 9) [40].

### 5.8. Saponins

Saponins are among the most challenging natural compounds for molecular docking because they combine a large hydrophobic aglycone with one or more carbohydrate chains.

Their high molecular mass and numerous rotatable bonds substantially restrict the applicability of conventional docking algorithms developed mainly for smaller drug-like molecules [18]. Accurate modeling of carbohydrate-fragment geometry is particularly difficult because standard force fields and scoring functions do not always describe the numerous hydrogen bonds and conformational mobility of sugar residues adequately [30,31,32].

Saponin docking results should therefore be treated as preliminary structural hypotheses requiring validation by molecular dynamics and binding-free-energy calculations (Section 9).

### 5.9. Polysaccharides

Polysaccharides largely fall outside the applicability domain of conventional molecular docking. Their high molecular mass, pronounced conformational variability, and numerous hydroxyl groups preclude a comprehensive description of binding using standard protocols designed for small organic molecules [18].

Accordingly, modeling studies usually examine oligosaccharide fragments containing the putative protein-interaction site rather than full-length polysaccharides. Even then, results depend strongly on the quality of conformer generation and accurate treatment of desolvation, which remains a major limitation of current scoring functions (Section 4 and Section 7) [30,31,32].

Interactions involving polysaccharides are preferably modeled using combined computational approaches integrating docking, molecular dynamics, and specialized carbohydrate-modeling methods.

### 5.10. Peptides

Natural peptides differ substantially from most classes discussed above because of the pronounced flexibility of their backbones and side chains [41]. These features complicate identification of the optimal binding pose and reduce the effectiveness of conventional docking algorithms.

The main limitation is the inability to account fully for conformational rearrangement of both the peptide and the target protein during binding, as discussed in Section 7. Programs developed for small organic molecules therefore provide satisfactory results mainly for short cyclic peptides.

Specialized protein-peptide docking methods followed by molecular dynamics are preferable for linear natural peptides and larger peptide ligands, allowing complex stability and interaction dynamics to be assessed (Section 9) [41].

### 5.11. Summary

Natural compounds cannot be treated as a homogeneous group of molecular docking objects. Their structural diversity, including differences in molecular mass, conformational mobility, number of stereogenic centers, functional groups, and ionization state, directly determines the appropriate computational protocol and materially affects result reliability.

Relatively compact and structurally rigid compounds, such as xanthones, coumarins, and many terpenoids, are the most favorable candidates for conventional molecular docking. Existing conformational-search algorithms and scoring functions generally provide satisfactory reproducibility for these compounds. By contrast, alkaloids, flavonoids, polyphenols, lignans, saponins, polysaccharides, and peptides require substantially more rigorous ligand preparation because of their protonation and tautomeric behavior, pronounced flexibility, or numerous rotatable bonds, as discussed in Section 3.3.

Most computational difficulties arise not from the docking algorithm itself but from ligand-structure preparation and result interpretation. Sources of systematic error differ among natural-product classes: protonation-state assignment is critical for alkaloids; treatment of multiple hydrogen bonds and desolvation effects for flavonoids and polyphenols; polycyclic scaffold geometry for terpenoids; and pronounced conformational mobility and violation of the applicability domain of standard docking algorithms for saponins, polysaccharides, and peptides.

A single computational protocol is not methodologically justified for all natural-product classes. Ligand preparation, docking parameters, interpretation of scoring functions, and the extent of downstream validation should reflect the structural characteristics of the compound under investigation. Consequently, the general limitations of molecular docking discussed in Section 7 manifest differently across natural-product classes.

These differences necessitate multilevel validation. Irrespective of chemical class, interpreting binding energies without redocking, interaction analysis, molecular dynamics, or free-energy calculations substantially increases the risk of false-positive conclusions. Molecular docking of natural compounds should be regarded primarily as an initial structure-based candidate-selection tool rather than independent evidence of biological activity. Subsequent validation, discussed in Section 9, is essential for reproducible and scientifically robust conclusions.

## 6. Major Protein Targets of Natural Compounds in Molecular Docking Studies

Protein-target selection is determined by the therapeutic focus of the study. Supplementary Table S1 summarizes both classical, historically well-characterized targets and contemporary targets that have entered docking practice over the past decade owing to advances in structural biology and cryogenic electron microscopy and the emergence of new therapeutic concepts, including targeting inflammasomes, the PI3K/AKT/mTOR pathway, metabolic receptors, and viral replication-complex proteins. A recommended PDB structure and the corresponding original structural publication are provided for each target [42,43,44,45,46,47,48,49,50,51,52,53,54,55,56,57,58,59,60,61,62,63,64,65,66,67,68,69,70,71,72,73,74,75,76,77,78,79,80,81,82,83,84,85,86,87,88,89,90,91,92,93,94,95,96,97,98,99,100,101,102,103,104,105,106,107,108,109,110].

### Major Trends in Protein-Target Selection

Analysis of the protein targets most frequently used in molecular docking studies of natural compounds reveals several general trends.

Therapeutic-target selection closely reflects the contemporary disease burden. Molecular docking studies of natural compounds have traditionally focused on cancer, cardiovascular, neurodegenerative, inflammatory, and infectious diseases. Oncological targets, including those relevant to breast cancer, are particularly prominent, reflecting the continuing interest in natural products as sources of anticancer lead structures [111].

First, contemporary docking is gradually shifting from relatively simple enzymatic systems towards substantially more complex protein assemblies. Early studies focused primarily on individual enzymes with well-characterized catalytic pockets, such as acetylcholinesterase, cyclooxygenase-2, and dihydrofolate reductase. More recent investigations increasingly address multidomain proteins, protein-protein interfaces, membrane receptors, transporters, and large signaling complexes, including inflammasomes and innate-immune proteins. This expansion has been enabled by advances in cryogenic electron microscopy, the availability of numerous high-quality experimental structures, and the use of AlphaFold models discussed in Section 3.1.

Second, interest has increased markedly in multifunctional signaling proteins that regulate several cellular processes simultaneously. In addition to classical enzymes, PI3K, AKT, mTOR, NLRP3, Keap1, TLR4, LRRK2, and other regulatory proteins implicated in cancer and inflammatory, neurodegenerative, and metabolic disorders are increasingly used as docking targets. Their pronounced conformational mobility restricts the applicability of conventional rigid-receptor docking and necessitates ensemble modeling or subsequent molecular dynamics (Section 7 and Section 9).

Third, the COVID-19 pandemic substantially altered the publication landscape of natural-product docking. Rapid determination of the three-dimensional structures of nearly all major SARS-CoV-2 proteins, including Mpro, PLpro, RdRp, NSP13, and NSP16, made the coronavirus one of the most extensively investigated systems for virtual screening of natural compounds. This development produced a marked increase in studies docking flavonoids, alkaloids, terpenoids, and other natural metabolites to viral proteins.

Another characteristic trend is the transition from searching for single-target compounds towards investigating the potential multitarget activity of natural products. Many natural metabolites can interact simultaneously with several proteins within the same signaling pathway or disease process. Contemporary studies therefore increasingly apply sequential docking against multiple targets and integrate molecular docking with network pharmacology and pathway analysis.

Protein-target selection directly determines the requirements of the computational protocol. Compact enzymatic active sites generally conform well to the assumptions of conventional molecular docking, whereas protein-protein interfaces, large allosteric sites, membrane receptors, and multiprotein complexes more often require advanced approaches such as ensemble docking, molecular dynamics, and binding free-energy calculations. Result reliability is therefore determined not only by the properties of the natural compound discussed in Section 5 but also by the structural organization of the protein target.

Advances in structural biology have thus substantially expanded the range of proteins accessible to molecular docking. At the same time, computational studies have become methodologically more complex, making appropriate experimental-structure selection, rigorous receptor preparation, and mandatory result validation increasingly important, as discussed in Section 3, Section 7 and Section 9.

## 7. Fundamental Limitations of Molecular Docking

Despite its widespread use in natural-product research, molecular docking remains an approximate computational model based on several physical and mathematical assumptions. Its high computational speed is achieved by substantially simplifying the actual protein-ligand system; consequently, even contemporary algorithms cannot reproduce molecular recognition in a living cell in full. Most limitations are fundamental and arise from the concept of conventional molecular docking rather than from any particular software package.

These limitations underlie most methodological errors identified in the analysis of published studies (Section 8). Reliable interpretation requires an understanding of the physical assumptions and applicability domain of the method before the computational experiment is initiated.

### 7.1. Limitations of the Protein Structural Model

Conventional molecular docking generally assumes that the protein can be represented as an essentially invariant three-dimensional structure. Most programs treat the receptor as rigid, permitting only restricted movement of selected side chains or excluding conformational changes entirely during ligand binding. Experimental evidence, however, shows that proteins exist as ensembles of interconverting conformations and that complex formation is often accompanied by local or global structural rearrangements [112].

This limitation is particularly pronounced for highly dynamic proteins, including kinases, G-protein-coupled receptors, transporters, multidomain enzymes, and protein-protein interfaces. In many cases, the active site forms only upon ligand binding or undergoes substantial induced-fit rearrangement, which conventional docking reproduces only partially. A single experimental structure may therefore fail to represent the full spectrum of possible interactions.

The quality of the structural model is an additional source of uncertainty. Even high-resolution PDB structures may contain missing polypeptide segments, engineered mutations, alternative side-chain conformations, or crystallization artifacts that affect binding-site geometry. Although these features were discussed in Section 3.1, they frequently determine the reliability of all subsequent modeling.

Structures predicted using artificial intelligence require separate consideration. AlphaFold has substantially increased the number of proteins available for structure-based modeling, but high accuracy of the overall fold does not guarantee comparable accuracy of local active-site geometry. This is particularly relevant to flexible loops and amino-acid side chains directly involved in ligand binding. When predicted structures are used, uncertainty in the receptor model therefore becomes an additional source of computational error.

Protein-structure selection should consequently be regarded as one of the most critical stages of a docking investigation. Errors introduced at this stage cannot be fully compensated for by contemporary pose-search algorithms or more sophisticated scoring functions.

### 7.2. Limitations of the Physical Model of the Protein-Ligand Complex

In addition to simplifying receptor structure, molecular docking employs a markedly simplified physical description of binding itself. Most current scoring functions primarily evaluate protein-ligand contact geometry, whereas several physical contributions are treated approximately or omitted entirely.

Solvent representation remains one of the most important limitations. Actual binding involves displacement of water molecules from the active site and reorganization of hydrogen-bonding networks, substantially affecting the free energy of the complex. Most algorithms, however, use implicit-solvent models or exclude water altogether and therefore account for desolvation only approximately. Polyphenols, glycosylated flavonoids, and other highly polar natural compounds are particularly sensitive to this issue because interactions with water are major determinants of their binding behavior.

Approximate treatment of entropy is equally important. Ligand binding reduces ligand conformational freedom, changes protein mobility, and redistributes solvent molecules. Conventional docking generally represents these processes using simplified empirical corrections or omits them completely. Calculated binding energies therefore do not constitute complete free energies of complex formation and may differ substantially from experimental measurements.

Metalloproteins create further difficulties. Coordination bonds involving zinc, magnesium, iron, and other metal ions possess partially covalent character and require specialized parameterization. Standard force fields do not always reproduce these interactions accurately, limiting docking accuracy for many metalloenzymes that constitute promising natural-product targets.

The computed binding energy is not an absolute physical quantity but an approximate estimate of relative complex stability within the selected model. Direct comparison of a docking score with experimental binding constants consequently requires considerable caution. This is a fundamental reason for discrepancies between computational and experimental results and explains the need for subsequent quantitative validation, discussed in Section 9.

### 7.3. Limitations of Contemporary Scoring Functions

Despite continued improvements in molecular docking algorithms, no current scoring function predicts binding energies with universal accuracy across different proteins and classes of natural compounds. As discussed in Section 4, available functions are based on distinct physical or statistical models and inevitably incorporate approximations that restrict their applicability.

A major challenge is the inability to describe simultaneously and accurately all factors governing protein-ligand complex formation. Most scoring functions reproduce ligand orientation reasonably well but predict absolute binding energies far less reliably. Large independent benchmarking studies have shown that even the best current algorithms yield only moderate correlations between calculated and experimental affinities, with performance depending strongly on both target type and ligand chemistry [18].

These limitations are especially apparent for natural compounds. Most algorithms were developed and validated primarily using libraries of synthetic drug-like molecules from PDBbind and related datasets. Large polyphenols, saponins, macrocycles, highly glycosylated compounds, and other atypical natural metabolites are markedly underrepresented in the training data, reducing the reliability of computational estimates.

Machine-learning methods introduce an additional source of uncertainty. Contemporary AI-based scoring functions can improve ranking for compounds resembling the training set, but their ability to extrapolate to new chemical classes remains limited. High accuracy on standard test sets therefore does not necessarily translate into comparable reliability for structurally unusual natural compounds.

Calculated binding energies should consequently be used primarily for relative comparisons among compounds within the same chemical series rather than as independent quantitative measures of biological activity. Misinterpretation of docking scores remains one of the most frequent errors in current publications, as confirmed by the analysis presented in Section 8.

### 7.4. Limitations Associated with Receptor and Ligand Preparation

Molecular docking quality depends not only on the software used but also on appropriate preparation of the protein structure and ligand. As demonstrated in Section 3, errors introduced during preparation are virtually impossible to correct through subsequent calculations.

For receptors, the main sources of uncertainty are selection of the experimental structure, treatment of water molecules, assignment of amino-acid protonation states, missing polypeptide segments, and representation of cofactors or metal ions. Even minor changes in active-site geometry can substantially alter the predicted binding pose.

Preparation of natural compounds is equally important. Incorrect protonation states, tautomeric forms, absolute configurations, or initial conformations can completely alter docking results before the computational experiment begins. These issues are particularly critical for alkaloids, flavonoids, saponins, and other compound classes discussed in Section 5.

The effect of structure preparation is often comparable to that of docking-program selection. Comparative assessments of protein- and ligand-preparation protocols have shown that modifying preprocessing parameters can substantially change virtual-screening outcomes even when the same docking algorithm is used [19].

Computational reproducibility depends less on the name of the software than on complete reporting of every receptor- and ligand-preparation step. Insufficient procedural detail severely restricts independent reproduction of published results and remains a common source of disagreement between studies.

### 7.5. Limitations in the Interpretation of Molecular Docking Results

Even when receptor and ligand preparation are appropriate, molecular docking results require cautious interpretation. The most common error is to equate a calculated docking score with experimental binding affinity. Scoring functions estimate only the relative likelihood of complex formation within a selected computational model and are not direct equivalents of experimental binding constants or interaction free energies.

Comparisons among structurally dissimilar natural compounds require particular caution. Because most scoring functions were calibrated primarily using relatively small drug-like molecules, absolute docking scores for different chemical classes are not necessarily comparable. The most defensible approach is to compare compounds within the same structural series using an identical computational protocol.

The absence of binding-kinetic information is another important limitation. Docking provides a static representation of the protein-ligand complex and cannot estimate the rate of complex formation, its stability over time, or ligand residence time in the active site. Such kinetic properties frequently determine in vivo pharmacological efficacy. Even the most energetically favorable predicted pose therefore does not guarantee a stable complex under biological conditions [113].

Computational reproducibility constitutes a separate challenge. Outcomes are influenced simultaneously by the quality of the initial protein structure, ligand-preparation parameters, search-region dimensions, scoring function, number of independent runs, and software implementation. Minor changes in these variables can alter compound ranking. Failure to report a detailed computational protocol therefore severely restricts independent verification of published results [114].

Many recent studies report only docking scores without adequately describing the parameters used, evaluating reproducibility, or independently validating the predicted complexes. This practice markedly reduces the scientific value of the computational experiment and precludes interpretation of the results as independent confirmation of the biological activity of a natural compound.

### 7.6. Section Summary

The limitations discussed above demonstrate that most molecular docking problems arise not from individual programs or scoring functions but from the fundamental assumptions of the method. A rigid receptor model, approximate treatment of solvent and entropy, limitations of current scoring functions, sensitivity to structure preparation, and ambiguity in interpreting calculated energies are systemic features of conventional molecular docking and affect all contemporary computational protocols to varying degrees.

A favorable docking score alone cannot be regarded as sufficient evidence of pronounced biological activity. Every docking result should be interpreted as a structural hypothesis requiring further evaluation using more rigorous computational and experimental methods.

An important question is the extent to which contemporary studies actually account for these limitations. Despite numerous methodological recommendations, their practical implementation has remained poorly characterized. The next section systematically examines original publications to quantify the prevalence of the most common methodological shortcomings in molecular docking studies of natural compounds.

## 8. Systematic Methodological Audit of Published Molecular Docking Studies

### 8.1. Study Objective and Cross-Model Audit with Independent Human Validation

This section constitutes a systematic methodological audit embedded within the broader narrative review. Its purpose was to quantify the reporting of selected practices that affect the interpretability and reproducibility of natural-product docking studies; it was not designed as a systematic review of biological efficacy, target-specific outcomes, or individual compound performance. The database searches were executed in August 2026, whereas the prespecified literature-coverage window was calendar year 2025. Publication year was assigned primarily from the electronic-publication (epub) date when available; records first published electronically in 2025 were retained even if a later 2026 issue assignment was present, whereas records first published electronically in 2026 were outside the target window. After eligibility screening and DOI deduplication, the final Stage 1 corpus contained 1127 unique publications.

A staged design was used to combine broad literature coverage with progressively more detailed verification. In Stage 1, all 1127 publications were evaluated using a structured coding framework by ChatGPT Work (OpenAI), powered by GPT-5.6 Sol. In Stage 2, a simple random sample of 80 publications was drawn exclusively from the same corpus and independently assessed from the available full texts by Claude Sonnet 5 (Anthropic). The second stage was intended to assess the consistency of the initial classifications and provide a higher-resolution description of reporting practice. A third verification layer consisted of an independent human assessment of 20 publications sampled without replacement from the 80-publication Stage 2 set (25% of that sample; random seed = 2026). The human reviewer applied the same seven operational definitions and recorded an independent classification for each criterion. The reviewer identity and article-level classifications are reported in Supplementary Table S4. Because the validation worksheet retained the corresponding Stage 2 fields for paired comparison, the human assessment is described as independent rather than formally blinded to the AI classifications. In addition, a targeted case-enriched validation set of 19 publications was assembled to test the two rare, interpretation-sensitive criteria—redocking and qualifying redocking RMSD < 2 Å—across positive, negative, and ambiguous Stage 2 records. Two human reviewers independently assessed both criteria, after which consensus classifications were recorded. Article-level judgments and supporting evidence are provided in Supplementary Table S5.

The use of two independently developed models reduced dependence on a single model architecture and provided a cross-model consistency check for large-scale coding. The additional human assessment provided a reference-standard check of the Stage 2 classifications in a prespecified random subsample. The AI-coded outputs are provided in Supplementary Tables S1 and S2, and the human-validation subsample and paired classifications are provided in Supplementary Table S4. The tools, versions, sampling procedure, and operational definitions are reported explicitly to support transparent reporting of AI-assisted evidence synthesis. Supplementary Table S5 provides the additional two-reviewer, case-enriched validation of redocking and redocking-RMSD coding.

Stage 1 describes the complete assembled corpus, Stage 2 provides an independent cross-model full-text audit of a random subset, and the 20-publication human subsample provides an additional expert verification layer. Estimates from the 1127-publication corpus (Supplementary Table S2) provide the broad methodological profile, whereas those from the 80-publication sample (Supplementary Table S3) provide a higher-resolution assessment with wider uncertainty intervals because of the smaller denominator. The human-validation analysis was used to assess agreement with Stage 2 rather than to generate a third prevalence estimate for the full corpus. The targeted S5 analysis was diagnostic rather than prevalence-estimating because its case-enriched composition was not intended to represent either the full corpus or the random Stage 2 sample.

### 8.2. Eligibility Criteria and Coding Framework

Eligible records were original peer-reviewed studies identified through Scopus or PubMed and falling within the prespecified 1 January–31 December 2025 publication window, defined by epub date when available and otherwise by the indexed publication date. Studies were required to dock one or more natural compounds to a protein target and to provide sufficient methodological information to classify the prespecified criteria. Reviews, conference abstracts, duplicate DOI records, exclusively ligand-based studies, and reports without assessable docking results were not eligible. Each publication was coded using explicit operational definitions; absence of an explicit statement in the available text was classified as not reported rather than assumed to be absent from the underlying research workflow.

The free-text validation-protocol field recorded the nature of additional computational or experimental evidence. Because it was descriptive and heterogeneous rather than a consistently binary variable, it was not pooled as a separate prevalence endpoint. The seven criteria in Table 2 formed a reporting profile used to calculate a seven-item reporting-completeness score and to assess cross-model agreement. The score measures breadth of reported methodological practices and should not be interpreted as an unweighted measure of scientific quality. The former shorthand label “positive control” is reported here as “known active ligand/reference comparator.” This category denotes contextual comparison with an established compound and must not be interpreted as equivalent to formal validation of pose reproduction, active–inactive discrimination, ranking, or affinity prediction. Equal weighting was chosen solely to provide a transparent descriptive count of reported items. A weighted score was not used because no externally validated weighting scheme exists for these heterogeneous criteria and because their relevance changes with the objective of the docking study. Assigning numerical weights would therefore introduce an unsupported hierarchy and could imply a degree of scientific-quality measurement that the audit was not designed to provide.

#### 8.2.1. Database Search Strategies and Rationale for Database Yields

The searches were performed in August 2026 to identify original natural-product molecular-docking studies within the prespecified 2025 publication window. The two databases were used to broaden literature coverage rather than to compare their retrieval performance. Because PubMed and Scopus use different indexing structures and field syntax, the search expressions were adapted to each database. The complete reproducible strategies were as follows:

PubMed: ((“molecular docking”[Title/Abstract] OR “docking study”[Title/Abstract] OR “docking studies”[Title/Abstract]) AND (“natural product”[Title/Abstract] OR “natural products”[Title/Abstract] OR “natural compound”[Title/Abstract] OR “natural compounds”[Title/Abstract] OR phytochemical*[Title/Abstract] OR flavonoid*[Title/Abstract] OR alkaloid*[Title/Abstract] OR terpenoid*[Title/Abstract] OR polyphenol*[Title/Abstract] OR xanthone*[Title/Abstract] OR coumarin*[Title/Abstract] OR lignan*[Title/Abstract] OR saponin*[Title/Abstract])) AND (“2025/01/01”[Date—Publication]: “2025/12/31”[Date—Publication]) AND free full text[sb].

Scopus: TITLE-ABS-KEY((“molecular docking” OR “docking study” OR “docking studies”) AND (“natural product*” OR “natural compound*” OR phytochemical* OR flavonoid* OR alkaloid* OR terpenoid* OR polyphenol* OR xanthone* OR coumarin* OR lignan* OR saponin*)) AND PUBYEAR = 2025 AND DOCTYPE(ar).

The difference in database yield should be interpreted in the context of these database-specific search conditions. PubMed and Scopus differ in journal coverage, indexing practices, date assignment, document-type classification, and implementation of field restrictions. PubMed was additionally restricted to records indexed as free full text because downstream article-level assessment required accessible source material, whereas Scopus used TITLE-ABS-KEY and article-type restrictions. This accessibility restriction was a pragmatic sampling decision rather than a claim that freely accessible literature is methodologically representative of all publications. Scopus consequently contributed 164 of 1384 identified records (11.8%) and 106 of 1144 eligible records before deduplication (9.3%). These proportions describe only the yield of the specified complementary search strategies and should not be interpreted as measures of the relative size, coverage, or quality of the two databases. A sensitivity search without the PubMed free-full-text restriction was not part of the prespecified audit and therefore cannot be used here to quantify the magnitude of accessibility-related selection. The query terms were centered on broad natural-product terminology and selected small-molecule classes; they did not explicitly include peptide*, oligopeptide*, polysaccharide*, or oligosaccharide*. Although records using broader terms could still be retrieved, studies indexed only under these omitted class names may have been missed.

#### 8.2.2. Reproducibility of AI-Assisted Classification

The AI-assisted assessment used a fixed article-level record structure consisting of the article title, DOI or other bibliographic identifier, and the retrieved article text available for assessment. Records were presented individually, and the models were instructed to use only evidence contained in the supplied record, without inferring an unreported method. Stage 1 used ChatGPT Work (OpenAI), powered by GPT-5.6 Sol; Stage 2 used Claude Sonnet 5 (Anthropic, San Francisco, CA, USA). Model names, versions, sampling procedures, operational definitions, and article-level outputs are reported so that the coding logic can be reapplied to the same source records. Supplementary Methods S1 reproduces the complete user-level instruction templates, input serialization format, output schema, operational definitions, ambiguity rules, and adjudication workflow. The platform-level system prompts used internally by the commercial services were not exposed to the authors and therefore cannot be reproduced; this constraint is stated explicitly rather than presenting an inferred or reconstructed system message as an exact prompt. This is a general limitation of closed, hosted commercial LLM platforms rather than a gap specific to this audit: researchers requiring complete prompt-level transparency should consider open-weight models with a fully inspectable prompting stack. Notably, the disclosed user-level protocol appears to have been the dominant determinant of the reported classifications, since Stage 1 and Stage 2 used different models operating under different, undisclosed system prompts yet achieved 98.3% agreement across 525 paired decisions (Section 8.7), and independent human review agreed with the AI classifications in 96.4% of decisions in the random validation subsample (Section 8.10).

The standardized instruction was: “Using only the supplied article text, classify each prespecified criterion as Yes, No, Unconfirmed, or N/A; provide the label and concise supporting evidence or location for each decision; do not count molecular-dynamics trajectory RMSD as redocking RMSD; and do not treat a known active/reference comparator as native-ligand redocking.” Yes was required when explicit evidence that the operational definition was met. No was used when the assessable article text was sufficient, and the procedure was not reported. Unconfirmed was retained when incomplete access, ambiguous wording, or insufficient evidence prevented a defensible binary decision. N/A was reserved for a criterion that was logically non-applicable, particularly redocking RMSD when no redocking assessment existed. Ambiguous responses were not silently forced into No; they were retained for paired comparison or referred to human review. Individual classifications are provided in Supplementary Tables S2–S5, including the human judgments and evidence used in the targeted S5 adjudication.

### 8.3. PRISMA 2020 Reporting of Study Flow

**Figure 1:**
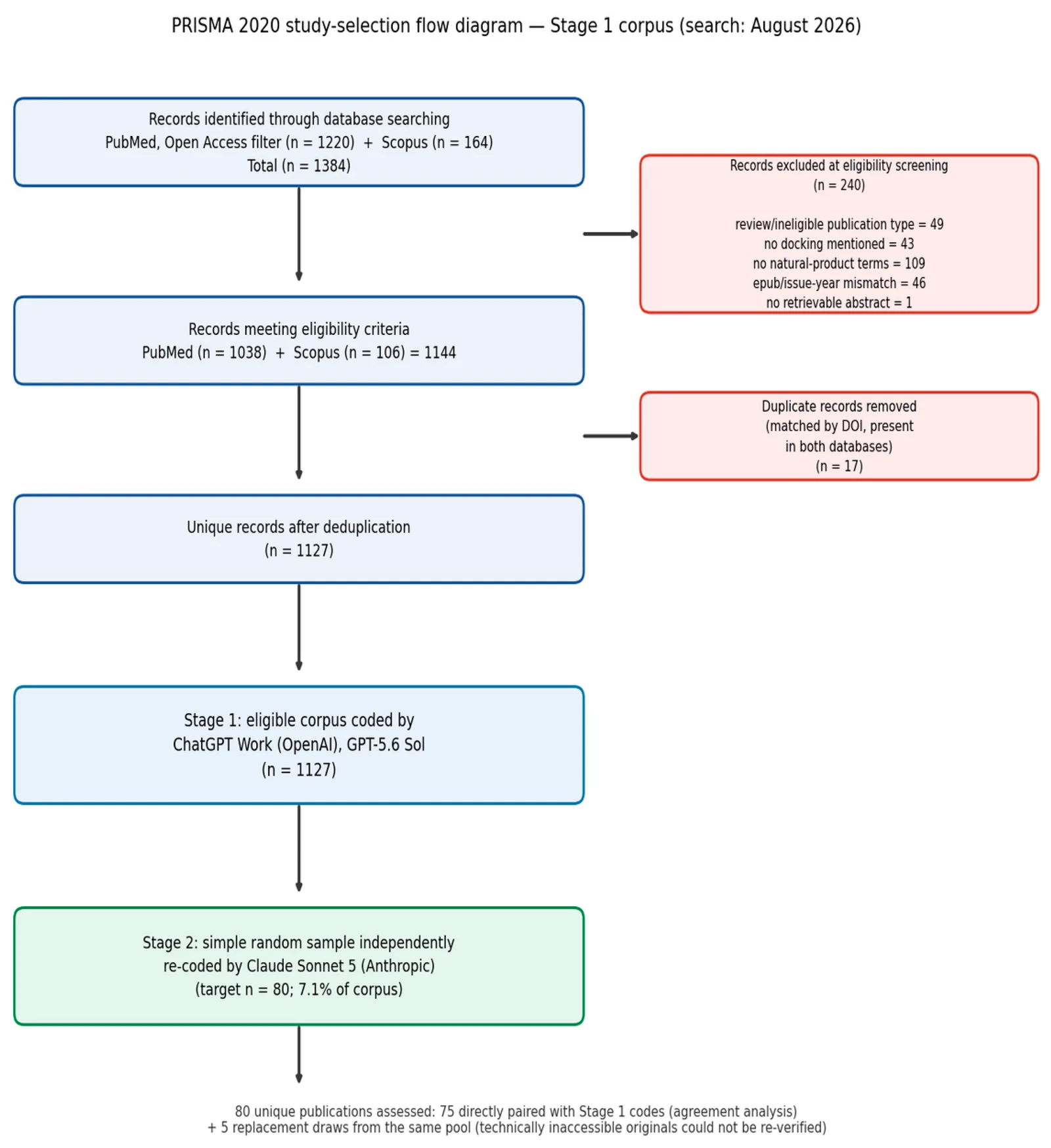
PRISMA-informed flow diagram for the two-stage cross-model methodological audit.

Study flow was documented according to PRISMA 2020 principles of transparent identification, eligibility assessment, inclusion, and record accounting (Figure 1). PubMed contributed 1220 records after application of the free-full-text filter, and Scopus contributed 164, giving 1384 identified records. Screening excluded 240 unique records, leaving 1038 eligible PubMed records and 106 eligible Scopus records (n = 1144). Removal of 17 duplicate DOI records present in both databases yielded the final Stage 1 corpus of 1127 unique publications. Exclusion-reason flags included ineligible publication type (n = 49), no explicit mention of molecular docking (n = 43), absence of natural-product terminology (n = 109), epub/indexed-year inconsistency with the 2025 coverage rule (n = 46), and no retrievable abstract (n = 1). Because these flags were not mutually exclusive, their sum exceeds the number of unique excluded records.

All 1127 records entered Stage 1 coding. A simple random sample of 80 records (7.1% of the corpus) was then selected from this pool for detailed Stage 2 assessment. Of these, 75 publications were directly paired with their Stage 1 classifications. Five originally sampled records could not be retrieved because of publisher paywalls or automated access restrictions and were replaced by random draws from the remaining records in the same corpus. Thus, all 80 publications contributed to the prevalence estimates in Table 3, whereas the 75 directly paired publications contributed to the cross-model agreement analysis in Table 4. For independent human validation, 20 publications were subsequently sampled without replacement from the final 80-publication Stage 2 set using a reproducible random seed of 2026. These 20 publications represented 25% of the Stage 2 sample and were assessed by a human domain-expert reviewer using the same seven criteria. A separate targeted set of 19 publications was subsequently used for case-enriched validation of redocking and redocking-RMSD classifications by two human reviewers. This diagnostic set intentionally included clear positive, clear negative, and potentially ambiguous records and was therefore analyzed separately from the random 20-publication seven-criterion validation sample.

### 8.4. Statistical Analysis

For every binary criterion, absolute frequency and proportion were calculated. Two-sided 95% Wilson score intervals were used because they remain stable for rare outcomes such as redocking and successful redocking RMSD. A seven-item reporting-completeness score was calculated as the unweighted number of criteria met (range, 0–7). For the 75 directly paired Stage 1/Stage 2 records, criterion-level cross-model agreement, the number and direction of discordant classifications, overall agreement across all 525 paired decisions, exact article-level profile agreement, and Cohen’s kappa were calculated. For the 20-publication human-validation subsample, the human classifications were compared with the corresponding Stage 2 classifications across 140 criterion decisions (20 publications × 7 criteria). Overall agreement, criterion-level agreement, the number and direction of discordant classifications, and exact seven-criterion article-profile agreement were calculated. Because the Stage 2 worksheet retained indeterminate labels such as Unconfirmed and N/A, these labels were preserved when characterizing disagreements rather than silently collapsing them into the binary No category. Cohen’s kappa was not emphasized for the human subsample because several rare criteria had invariant or highly unbalanced marginal distributions. The analysis was descriptive; no null-hypothesis significance tests were used. Differences between the full corpus and the random Stage 2 sample are reported in percentage points and interpreted as an audit comparison rather than as evidence of temporal or causal effects. For the targeted 19-publication analysis, agreement between the two human reviewers was summarized across 38 decisions (19 publications × 2 criteria). Their consensus classifications were then compared with Stage 2 using overall decision-level agreement, criterion-level agreement, the number and direction of revisions, and exact two-criterion article-profile agreement. No prevalence estimate was derived from this deliberately enriched set. The unweighted score was prespecified for descriptive simplicity and reproducibility, not because all seven criteria were assumed to have equal scientific importance.

### 8.5. Results in the 1127-Publication Corpus (Stage 1, ChatGPT Work)

**Figure 2:**
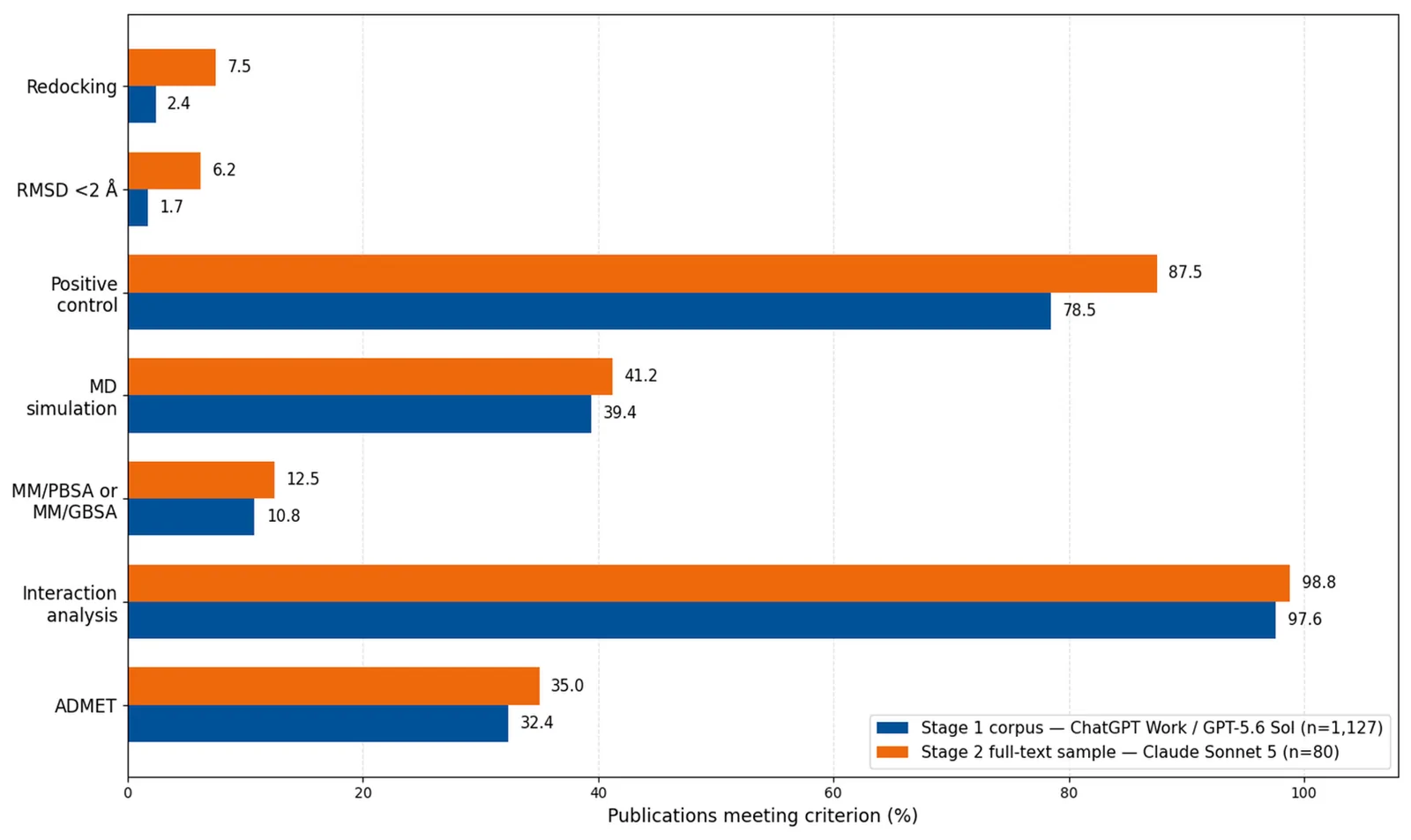
Criterion frequencies in the ChatGPT-coded corpus (Stage 1) and the Claude-coded full-text sample (Stage 2). The abbreviated figure label “Positive control” corresponds to the category “known active ligand/reference comparator”.

All 1127 records had an identified full-text source in the analytic table (Supplementary Table S2). Interaction analysis was the most frequently documented component, appearing in 1100 publications (97.6%; 95% CI, 96.5–98.3%). Positive controls were reported in 885 studies (78.5%; 95% CI, 76.0–80.8%). Molecular dynamics was used in 444 studies (39.4%; 95% CI, 36.6–42.3%), ADMET assessment in 365 (32.4%; 95% CI, 29.7–35.2%), and MM/PBSA or MM/GBSA in 122 (10.8%; 95% CI, 9.1–12.8%). Internal pose-reproduction checks were rare: redocking was reported in 27 publications (2.4%; 95% CI, 1.7–3.5%), and a redocking RMSD below 2 Å was documented in 19 (1.7%; 95% CI, 1.1–2.6%) (see Figure 2).

The mean reporting-completeness score in the corpus was 2.63 of 7 criteria, and the median was 2. The most common score was 2, observed in 500 studies (44.4%), followed by a score of 3 in 300 (26.6%) and 4 in 148 (13.1%). Ninety-seven studies (8.6%) met one criterion and 12 (1.1%) met none. Only seven publications (0.6%) met all seven criteria; a further nine (0.8%) met six. Nineteen publications (1.7%) jointly reported redocking and RMSD <2 Å. Among the 444 studies using MD, 118 also reported MM/PBSA or MM/GBSA, corresponding to 26.6% of MD studies and 10.5% of the full corpus. These distributions quantify only the number of prespecified procedures reported. They do not imply that a study reporting more items is necessarily scientifically superior, because the appropriate methodological set depends on the study objective, available structural information, and strength of the claims made.

### 8.6. Results of the 80-Publication Full-Text Audit (Stage 2, Claude Sonnet 5)

The independent full-text audit reproduced the overall hierarchy of methods observed in Stage 1. Interaction analysis was identified in 79 of 80 publications (98.8%; 95% CI, 93.3–99.8%), positive controls in 70 (87.5%; 95% CI, 78.5–93.1%), MD in 33 (41.2%; 95% CI, 31.1–52.2%), ADMET in 28 (35.0%; 95% CI, 25.5–45.9%), and MM/PBSA or MM/GBSA in 10 (12.5%; 95% CI, 6.9–21.5%). Redocking was confirmed in six studies (7.5%; 95% CI, 3.5–15.4%), while five (6.2%; 95% CI, 2.7–13.8%) documented RMSD < 2 Å. Five publications met both redocking and RMSD criteria, and one reported redocking without a qualifying RMSD value.

The audited sample showed higher proportions than the complete corpus for each criterion: redocking (+5.1 percentage points), RMSD < 2 Å (+4.5), positive controls (+9.0), MD (+1.9), MM/PBSA or MM/GBSA (+1.7), interaction analysis (+1.2), and ADMET (+2.6). These differences may reflect both sampling variability and the greater sensitivity of detailed full-text assessment. They should therefore be interpreted as an audit comparison rather than as bias-corrected prevalence estimates for the complete corpus. For interpretation of methodological frequencies, the Stage 2 estimates are therefore given greater evidentiary weight because they derive from detailed assessment of the available full texts. The Stage 1 estimates remain useful as broad screening-level descriptors of the assembled corpus, but the systematic direction of discordance indicates under-detection at that stage, particularly for rare criteria.

**Figure 3:**
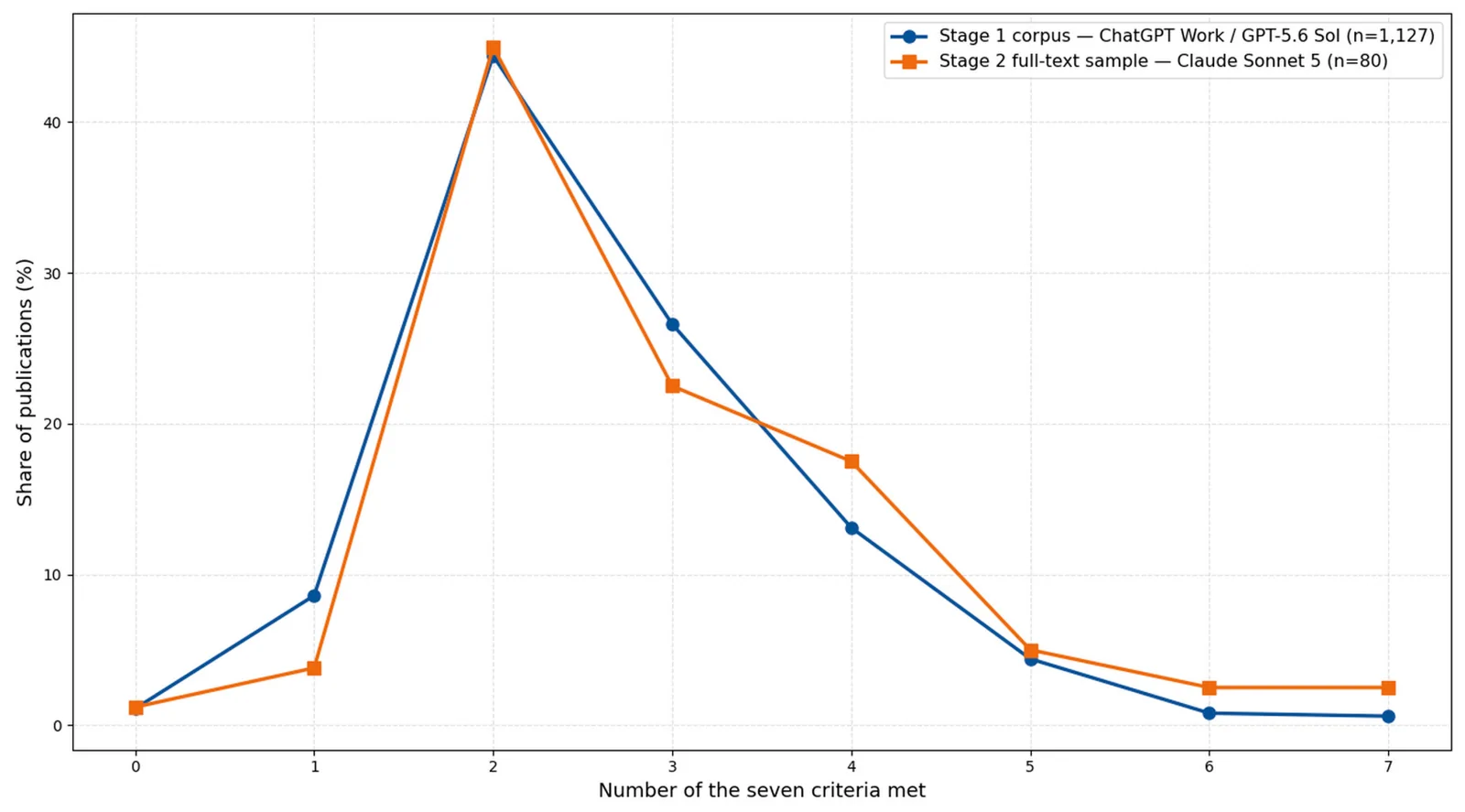
Distribution of the seven-item methodological reporting-completeness score in the Stage 1 corpus and the Stage 2 sample.

The mean completeness score in the sample was 2.89, and the median was 2.5. Thirty-six publications (45.0%) met two criteria, 18 (22.5%) met three, and 14 (17.5%) met four. Two publications (2.5%) met all seven criteria, two (2.5%) met six, and one (1.2%) met none. All 10 sample publications reporting MM/PBSA or MM/GBSA also reported MD; this corresponds to 30.3% of the 33 MD studies. The score is therefore interpreted as reporting breadth rather than a validated scale of methodological or scientific quality (see Figure 3).

### 8.7. Cross-Model Agreement: ChatGPT Stage 1 Coding Versus Claude Stage 2 Full-Text Audit

**Figure 4:**
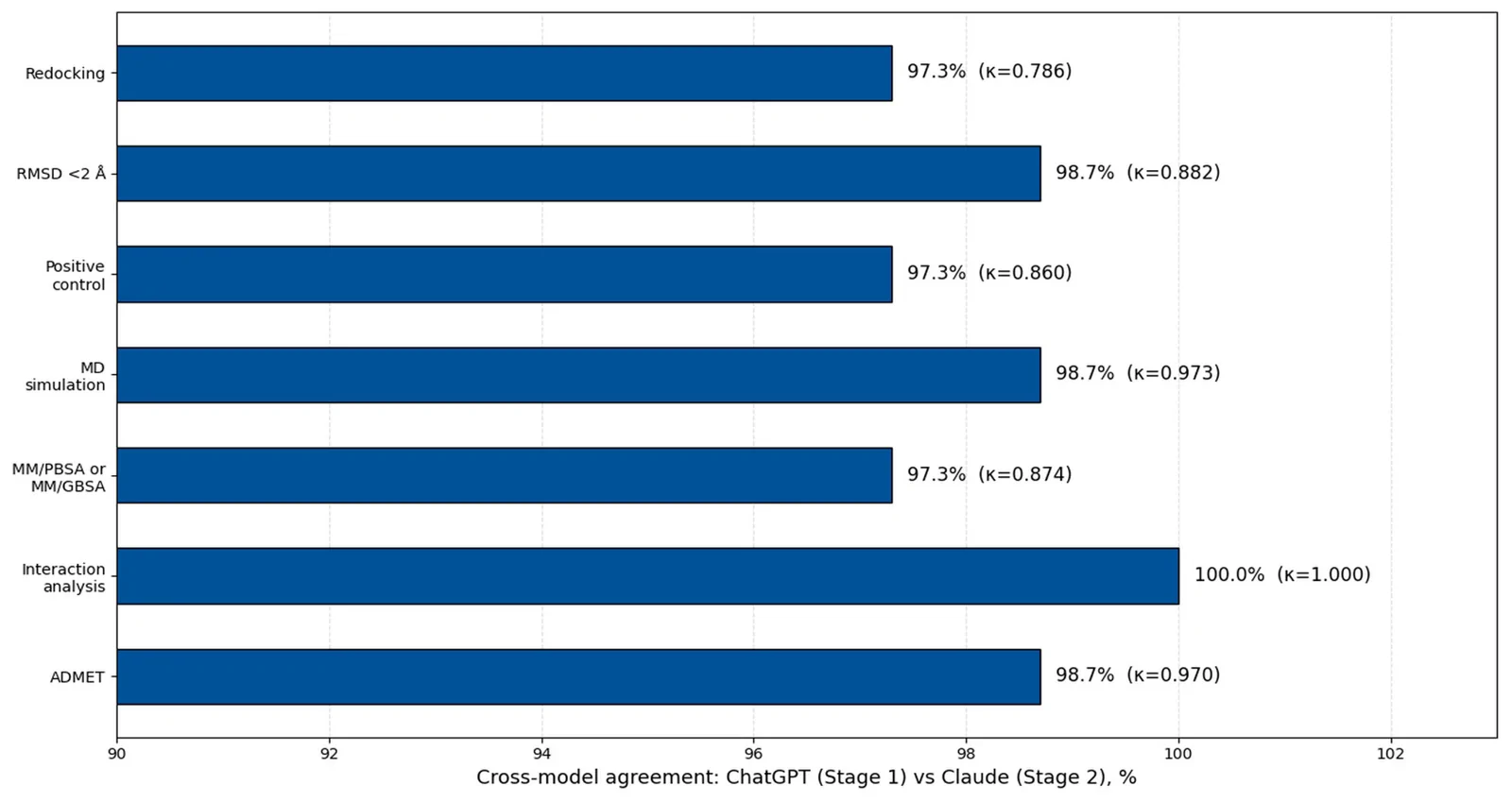
Criterion-level cross-model agreement between ChatGPT Stage 1 coding and the Claude Sonnet 5 full-text audit. The abbreviated figure label “Positive control” corresponds to the category “known active ligand/reference comparator”.

Agreement was evaluated for the 75 publications with directly paired Stage 1 and Stage 2 classifications. Across the seven criteria, 516 of 525 binary decisions agreed (98.3%), and exact agreement across the complete seven-criterion profile was obtained for 68 of 75 publications (90.7%). Nine discordant decisions occurred in seven publications. In every case, a criterion not detected during Stage 1 was identified during the Stage 2 full-text assessment; no Stage 1 positive classification was subsequently reversed. The observed discrepancies therefore reflected under-detection during Stage 1 rather than over-classification (see Figure 4).

Agreement ranged from 97.3% for redocking, positive-control, and MM/PBSA or MM/GBSA coding to 100.0% for interaction analysis. Cohen’s kappa ranged from 0.786 for redocking to 1.000 for interaction analysis. The comparatively lower kappa values for redocking and positive control, despite high raw agreement, reflect kappa’s sensitivity to unbalanced marginal distributions rather than a materially higher error rate: because most publications are negative for redocking, a small number of discordant cells has a proportionally larger effect on kappa than on simple percentage agreement. Raw percentage agreement is also prevalence sensitive: when a criterion is rare, agreement on the many negative classifications can yield a high percentage even if some positive cases are missed. Accordingly, the 98.3% overall agreement is interpreted together with criterion prevalence, Cohen’s kappa, the direction of discordance, and the Stage 2 full-text frequencies rather than as stand-alone evidence of equivalent detection performance.

### 8.8. Independent Human-Expert Validation of the Stage 2 Classifications

Twenty publications were randomly selected without replacement from the 80-publication Stage 2 sample for independent assessment by a human reviewer. The same operational definitions were applied to redocking, qualifying redocking RMSD < 2 Å, positive control, MD simulation, MM/PBSA or MM/GBSA, interaction analysis, and ADMET. The reviewer identity and paired article-level classifications are reported in Supplementary Table S4. Overall, the human classifications agreed with the Stage 2 classifications in 135 of 140 criterion decisions (96.4%). Five discordant decisions occurred in three publications: publication No. 151, RMSD < 2 Å (AI: N/A; human: No); publication No. 401, MD simulation (AI: Unconfirmed; human: No) and MM/PBSA or MM/GBSA (AI: Unconfirmed; human: No); and publication No. 847, positive control (AI: Unconfirmed; human: No) and ADMET (AI: Unconfirmed; human: No). Criterion-level agreement was 20/20 (100%) for redocking and interaction analysis and 19/20 (95.0%) for RMSD < 2 Å, positive control, MD simulation, MM/PBSA or MM/GBSA, and ADMET. Exact agreement across the complete seven-criterion article profile was obtained for 17 of 20 publications (85.0%).

All five disagreements had the same methodological direction: the AI-coded Stage 2 assessment retained an indeterminate or non-confirmed category (Unconfirmed, or N/A for the RMSD item in publication No. 151), whereas the human reviewer resolved the item as No. No disagreement involved an AI-positive classification being rejected by the human reviewer, or a human-positive classification missed by Stage 2. This pattern is informative because it indicates that the residual disagreement arose primarily at the boundary between an explicit negative classification and insufficient or non-applicable evidence, rather than from contradictory judgments about clearly reported positive methods. In practical terms, the human reviewer applied a more definitive binary interpretation to several borderline records, whereas the AI-assisted extraction preserved a three-state distinction between Yes, No, and indeterminate/non-confirmed evidence. The human-validation subset contained no positive redocking classifications and no qualifying redocking RMSD values; therefore, the high agreement should not be interpreted as evidence of sensitivity for detecting positive cases for these rare criteria. The paired human and Stage 2 classifications are reported in Supplementary Table S4.

### 8.9. Targeted Case-Enriched Human Validation of Redocking and Redocking-RMSD Classification

The targeted analysis comprised 19 publications selected to challenge classification of redocking and qualifying redocking RMSD < 2 Å, including clear positive, clear negative, and ambiguous Stage 2 records (Supplementary Table S5). Reviewer 1 and Reviewer 2 independently produced identical classifications for every item, corresponding to 38/38 agreement (100%) across the two criteria. The resulting consensus identified redocking in 8 of 19 publications and a qualifying redocking RMSD in 6 of 19. These proportions describe the enriched validation set only and must not be interpreted as prevalence estimates.

Compared with Stage 2, the human consensus agreed in 31 of 38 criterion decisions (81.6%): 15/19 (78.9%) for redocking and 16/19 (84.2%) for RMSD < 2 Å. Seven decisions were revised across four publications, and exact agreement for the complete two-criterion profile was obtained for 15/19 publications (78.9%). Three redocking-positive and two RMSD-positive classifications were identified by the human reviewers but not confirmed at Stage 2, whereas one Stage 2 redocking-positive and its associated RMSD-positive classification were rejected after human review. The revisions arose chiefly from distinguishing native-ligand redocking from docking of positive controls or visual pose alignment, and from separating redocking-pose RMSD from RMSD reported for molecular-dynamics trajectories. This targeted exercise therefore exposed classification-boundary errors that were not represented in the original random human-validation subset.

### 8.10. Interpretation and Limitations

The staged methodological audit demonstrates a consistent pattern. Descriptive interaction mapping and the use of reference comparators were common, whereas direct validation of docking-pose recovery was exceptional. MD was included in approximately two-fifths of studies, but binding free-energy calculations were used in only about one-tenth. The result is a marked imbalance between methods that describe a predicted complex and methods that directly test docking-protocol performance or provide complementary assessment of the resulting complex.

The independent Stage 2 full-text audit supports the stability of this overall pattern. Stage 2 identified several additional instances of redocking, positive controls, MD, MM/PBSA or MM/GBSA, and ADMET, but the ranking of criteria remained unchanged and redocking with a qualifying RMSD remained uncommon. Agreement across the 525 Stage 1/Stage 2 paired decisions was high (98.3%), indicating strong cross-model consistency under explicit operational definitions. The subsequent human validation agreed with Stage 2 in 135 of 140 criterion decisions (96.4%), with exact seven-criterion profile agreement in 17 of 20 publications (85.0%). Importantly, all five human-versus-Stage 2 discrepancies concerned AI-coded indeterminate/non-confirmed labels that the human reviewer resolved as No; none involved disagreement over a clearly positive classification. This human comparison therefore supports the overall consistency of the Stage 2 coding while also identifying a specific classification-boundary issue that should be considered when AI-assisted evidence extraction uses more than two response states. The complementary case-enriched analysis sharpened this interpretation: although the two human reviewers agreed completely with one another, consensus-versus-Stage 2 agreement fell to 81.6% for the deliberately challenging redocking/RMSD decisions. Thus, high agreement in a random sample can coexist with lower accuracy at rare, semantically difficult classification boundaries. Because every Stage 1/Stage 2 discordance represented a Stage 2 detection missed at Stage 1, the detailed 80-publication results provide the more informative frequency estimates for interpretation, while the 1127-publication results are retained as screening-level estimates of the assembled corpus.

The cross-model and human comparisons nevertheless require cautious interpretation. All nine Stage 1/Stage 2 discordant decisions involved criteria identified at Stage 2 but not at Stage 1, a pattern consistent with under-detection during the larger-scale first-stage assessment. In the human-validation subset, the five discordant decisions instead reflected how borderline evidence was encoded: Stage 2 preserved Unconfirmed or N/A, whereas the human reviewer assigned No. Thus, the observed 96.4% agreement should not be interpreted as proof of error-free AI extraction; rather, it indicates high consistency accompanied by a reproducible semantic difference in handling indeterminate evidence. The 20-publication subset also contained no positive redocking or qualifying redocking-RMSD cases and was not designed to estimate sensitivity for these rare outcomes. The S5 results address this gap by including positive and ambiguous cases, but their case-enriched design precludes extrapolation of the observed 81.6% agreement or the consensus-positive proportions to the full corpus. They should instead be interpreted as a targeted stress test demonstrating the need for explicit evidence rules and human adjudication when distinguishing true native-ligand redocking and redocking-pose RMSD from related but non-equivalent procedures.

Several additional limitations should be considered. The corpus reflects searches performed in August 2026 for a prespecified 2025 publication window and is not a census of all natural-product docking research. PubMed was restricted by a free-full-text filter to facilitate downstream assessment, whereas Scopus used a field-restricted TITLE-ABS-KEY query and article-type filtering. The smaller Scopus yield (164 identified records versus 1220 from PubMed) therefore reflects database coverage together with the specific indexing, field syntax, and retrieval constraints used here, rather than an intrinsically smaller body of relevant literature in Scopus. Subscription-only literature and records not captured by the specified natural-product terminology may consequently be under-represented. In addition, reporting was used as a proxy for methodological practice, so an unreported procedure may have been performed but omitted from the article or Supplementary Materials. The seven-item score assigns equal weight to different practices and therefore measures completeness of reporting rather than validated scientific quality. The 80-publication audit has wider confidence intervals for rare outcomes, and five replacement draws were required when originally sampled records could not be retrieved. In a small number of technically inaccessible cases, assessment relied partly on detailed abstracts, indexed text, or publisher snippets rather than complete full text, potentially reducing sensitivity for methods reported only in Methods or Supplementary sections. Finally, the human validation comprised 20 publications (25% of the Stage 2 sample) and yielded 135/140 agreement (96.4%). The five disagreements show that agreement depends partly on how indeterminate AI labels are mapped to a binary reporting framework. Rare positive findings—particularly redocking and qualifying redocking RMSD—were not represented in this subset, and additional human validation enriched for such cases would further strengthen estimates of classification performance. The targeted S5 validation was limited to 19 publications and two criteria; although complete agreement between its two reviewers strengthens the internal consistency of the consensus, selection enrichment means that the 31/38 consensus-versus-Stage 2 agreement is diagnostic of difficult cases rather than an unbiased estimate of AI classification performance. The asymmetric accessibility restriction—free full text in PubMed but no equivalent filter in Scopus—may also have shifted the analytic corpus toward openly accessible literature and limits its representativeness. Moreover, because peptides and polysaccharides were discussed in the narrative review but were not explicit search terms, the audit should not be interpreted as a complete survey of those classes. The quantitative conclusions are therefore restricted to publications captured by the prespecified database fields, terminology, date window, and accessibility criteria, rather than generalized to all natural-product docking literature. A further limitation is that the unweighted completeness score treats each reported item as one unit despite substantial differences in its relevance to docking validation. For example, redocking directly examines pose reproduction, whereas ADMET addresses developability rather than docking accuracy. The score should therefore be read only as a count of reported components, not as a weighted index of validation strength or study quality. A further limitation concerns prompt-level reproducibility: ChatGPT Work and Claude are closed, hosted commercial services, and their platform-level system prompts are not exposed to users and could not be disclosed here. We report the complete user-level instruction templates, operational definitions, and classification rules that were within our control (Supplementary Methods S1), but full prompt-level transparency was not achievable and remains a general constraint of auditing methodology with closed commercial LLM platforms rather than open-weight, fully inspectable models.

Overall, the analysis identifies internal docking validation as the main methodological deficit. A minimum reproducible protocol should therefore prioritize native-ligand redocking with numerical RMSD, an explicit positive comparator, complete parameter reporting, and a clear distinction between docking-pose RMSD and trajectory RMSD. MD, MM/PBSA or MM/GBSA, ADMET, and interaction analysis provide valuable complementary evidence but cannot substitute for direct validation of the docking procedure itself.

## 9. Practical Recommendations for Validating Molecular Docking Studies of Natural Compounds

### 9.1. The Need for Multilevel Validation

Molecular docking is an effective tool for initial virtual screening; however, as demonstrated in Section 7, its results are affected by numerous sources of systematic error arising from limitations of the protein structural model, approximate descriptions of physical interactions, scoring-function performance, and receptor and ligand preparation. A favorable docking score alone therefore cannot be regarded as sufficient evidence that a natural compound binds strongly to the protein target under investigation. A docking score is an algorithm-specific ranking surrogate; binding affinity is an experimentally measurable thermodynamic property; and biological activity additionally reflects exposure, cellular context, off-target effects, and downstream mechanisms. These quantities must not be used interchangeably.

The methodological audit in Section 8 showed that most contemporary studies were limited to basic molecular docking, whereas comprehensive verification of the computational protocol was considerably less common. In the random full-text sample, redocking was reported in 7.5% of studies, a redocking RMSD below 2 Å in 6.2%, molecular dynamics in 41.2%, and MM/PBSA or MM/GBSA calculations in 12.5%. These findings reveal a substantial gap between current methodological recommendations and actual docking practice.

A contemporary docking study of natural compounds should be viewed as a sequence of interdependent validation steps. Each successive stage addresses limitations of the preceding stage and progressively increases the reliability of computational conclusions.

### 9.2. First Validation Level: Internal Verification of Docking-Protocol Performance

Internal validation of the computational protocol should constitute the minimum quality-control step in any docking study. Its primary purpose is to establish whether the selected software, search parameters, and scoring function can reproduce an experimentally determined protein-ligand complex.

The most widely used procedure is redocking, in which the co-crystallized ligand is removed from the experimental protein structure and redocked into the same active site. The predicted pose is then compared with the experimental pose by calculating the root-mean-square deviation (RMSD) of atomic coordinates. An RMSD of ≤2.0 Å is commonly regarded as evidence of successful pose reproduction, although an appropriate threshold may depend on the target and the resolution of the experimental structure [18].

Redocking and RMSD calculation should be treated as a single internal quality-assurance step. Redocking without a quantitative RMSD assessment does not permit objective evaluation of pose reproduction. Conversely, a low RMSD shows only that the protocol can reproduce a known complex and does not guarantee accurate predictions for new compounds. It does not demonstrate active–inactive discrimination, enrichment in virtual screening, correct ranking of a ligand series, quantitative affinity prediction, or biological activity; each of these objectives requires a different benchmark.

Internal validation is necessary but insufficient. It supports the appropriateness of the selected modeling parameters but does not establish complex stability, account for protein dynamics, or provide the actual binding free energy.

The 2 Å criterion is a conventional heuristic rather than a universal pass–fail boundary. It is most readily interpreted for relatively small, rigid ligands with unambiguous atom correspondence. For bulky or highly flexible natural products—including macrocyclic peptides, saponins, and oligomeric polyphenols—a single whole-ligand heavy-atom RMSD may be inflated by peripheral-group motion, multiple symmetry-equivalent atom mappings, or alternative orientations that preserve the same key pharmacophoric contacts. Conversely, a low global RMSD can conceal an incorrect orientation of a functionally important moiety.

For these ligands, the RMSD definition, atom mapping, symmetry treatment, and threshold should be specified before analysis and justified for the ligand class and study objective. Whole-ligand RMSD should be interpreted together with visual pose inspection, core- or scaffold-aligned RMSD, per-fragment displacement where appropriate, recovery of key protein–ligand contacts, and consistency with experimental binding-site information. A threshold above 2 Å may be defensible for a prespecified flexible-ligand protocol, but it should not be selected retrospectively to convert an unsuccessful redocking result into a positive one.

### 9.3. Second Assessment Level: Evaluation of Complex Stability by Molecular Dynamics

Redocking establishes whether a computational protocol can reproduce an experimentally known binding pose but does not assess its stability over time. As discussed in Section 7, conventional docking provides a static representation of the protein-ligand complex and does not capture the intrinsic conformational mobility of the protein, ligand, and surrounding solvent. Molecular dynamics (MD) can therefore constitute a subsequent complementary assessment stage when dynamic stability or receptor flexibility is relevant to the scientific question.

Unlike docking, MD simulates the temporal evolution of the complex, allowing retention of the predicted pose, persistence of key interactions, and potential receptor rearrangements to be assessed. Rapid departure of the ligand from the binding pocket or a major change in its initial orientation indicates that the original docking result may be unreliable, even when the docking score is favorable [112].

An MD trajectory should be evaluated using a set of complementary stability indicators rather than a single parameter. Particularly informative measures include the time evolution of complex RMSD, residue-level root-mean-square fluctuation (RMSF), persistence of key hydrogen bonds and hydrophobic contacts, and the ligand interaction pattern within the active site. Integrated interpretation of these descriptors provides a more reliable assessment of complex stability than static docking alone. RMSD calculated along an MD trajectory measures time-dependent structural deviation from a chosen reference and is conceptually distinct from redocking RMSD, which compares a predicted ligand pose with its crystallographic pose.

Molecular dynamics does not validate a docking pose in the strict sense; rather, it provides a complementary assessment of the dynamic behavior of the proposed complex. Docking generates a hypothesis regarding the three-dimensional organization of a complex, whereas MD tests the stability of that hypothesis using a more realistic physical model.

Despite this central role, molecular dynamics was used in 41.2% of studies in the Stage 2 full-text sample (33/80); the corresponding Stage 1 corpus estimate was 39.4% (444/1127).

### 9.4. Third Assessment Level: Approximate Estimation of Binding Free Energy

Even an MD-stable complex does not directly provide a quantitative binding free energy. Post-docking free-energy methods are required for this purpose, with MM/GBSA and MM/PBSA being the most widely used approaches.

Unlike conventional scoring functions, MM/GBSA and MM/PBSA evaluate ensembles of structures, commonly sampled from molecular-dynamics trajectories, and include molecular-mechanical and implicit-solvation terms [115]. In some systems and carefully controlled congeneric series, these methods may improve relative ranking compared with an initial docking score; however, improved agreement with experiment is not guaranteed and should not be assumed as a general property.

The accuracy of these endpoint methods depends strongly on the quality and sampling of the preceding MD trajectory and on methodological choices such as dielectric treatment and entropy approximation. MM/GBSA and MM/PBSA should therefore be regarded as approximate, model-dependent estimates of relative binding energetics rather than definitive quantitative validation of binding. Their performance is system-dependent and sensitive to conformational sampling, force-field parameters, trajectory length, dielectric treatment, treatment of water and ions, and whether entropic contributions are included.

Free-energy perturbation (FEP) remains the most rigorous computational approach to estimating binding-energy differences [116,117], but its high computational cost restricts routine application. FEP is currently used mainly for final ranking of a small number of structurally related compounds after virtual screening, whereas MM/GBSA and MM/PBSA provide a more practical compromise between computational cost and accuracy.

Published studies use MM/GBSA and MM/PBSA considerably less frequently than molecular dynamics. In the random full-text sample analyzed in Section 8, binding free-energy calculations were reported in 12.5% of publications compared with 41.2% for molecular dynamics, demonstrating the limited use of these informative complementary energetic-assessment methods.

### 9.5. Fourth Validation Level: Experimental Confirmation

Despite substantial advances in computational methodology, no available approach can fully replace experimental confirmation. Molecular docking, molecular dynamics, and free-energy calculations increase the likelihood of identifying promising compounds, but all remain tools for generating and testing computational hypotheses.

The most convincing confirmation of docking results is provided by experiments that directly measure ligand-target interactions. These include enzyme assays and determination of binding constants by isothermal titration calorimetry (ITC), surface plasmon resonance (SPR), microscale thermophoresis (MST), and related biophysical methods. Unlike cell-based assays, these approaches can confirm direct interaction between the compound and the proposed molecular target.

Experimental testing should constitute the final stage of comprehensive computational validation, particularly in studies claiming the discovery of new lead compounds.

### 9.6. Recommended Protocol for Molecular Docking Studies of Natural Compounds

On the basis of current methodological recommendations and the present analysis, we propose the practical protocol for molecular docking studies of natural compounds summarized in Table 5. The protocol is conditional on the structural information available for a given target and is intended to reduce false-positive interpretation rather than to prescribe an identical workflow for every system.

The proposed protocol reflects a contemporary sequence of computational assessment but does not require every stage to be performed in every study. Experimental structures and native-ligand redocking should be preferred when suitable reference complexes are available. When no appropriate co-crystallized ligand or experimental receptor structure exists, the limitation should be stated explicitly, and alternative validation strategies should be used where possible, including benchmarking against known active/inactive ligands, comparison with experimentally characterized binding-site residues, ensemble or predicted-structure assessment, and subsequent experimental testing. Validation should therefore be selected according to the study objective rather than applied as a universal checklist. When no co-crystallized ligand is available, benchmarking against experimentally characterized active and inactive compounds is especially important for screening or ranking claims.

The methodological audit showed that these basic procedures are currently omitted most frequently, whereas additional approaches such as ADMET and interaction analyses are used considerably more often (Section 8). This pattern indicates a shift in emphasis from validating the computational model towards describing its outputs. Interaction analysis assists structural interpretation but does not validate sampling or scoring, while ADMET addresses pharmacokinetic and toxicological properties rather than docking accuracy.

Table 6 links common docking objectives to the validation evidence that directly addresses each claim. Interaction mapping and ADMET assessment are included as complementary analyses: interaction analysis supports structural interpretation, whereas ADMET concerns pharmacokinetic and toxicological developability; neither validates docking-protocol performance.

### 9.7. Practical Recommendations for Authors

The analysis supports several practical recommendations for improving reporting transparency and fit-for-purpose methodological rigor in molecular docking studies of natural compounds.

First, every study should describe protein and ligand preparation in detail, including protonation states, the PDB structure used, search-space parameters, and software version.

Second, internal verification by native-ligand redocking with numerical RMSD should be performed when a suitable experimentally determined reference complex is available. When redocking is not feasible, the study should explain why and use an alternative benchmark appropriate to the target and available experimental evidence.

Third, docking results should not be interpreted solely from docking scores. Pose prediction, compound ranking, affinity estimation, and biological activity are distinct endpoints and should not be treated as interchangeable. Evaluation should include the three-dimensional organization of the complex and the interaction pattern; when appropriate, molecular dynamics can provide a complementary assessment of dynamic stability.

Finally, MM/GBSA or MM/PBSA may provide approximate relative energetic estimates for selected complexes, but these methods remain model-dependent. Definitive claims of direct target engagement or biological activity should be supported by independent experimental evidence whenever such claims are central to the study.

### 9.8. Summary

Contemporary molecular docking should be regarded not as an independent method for proving biological activity but as an initial structure-based approach for generating hypotheses about ligand-target recognition. Pose prediction, ranking accuracy, affinity prediction, and biological activity represent distinct levels of inference. Computational reliability depends on appropriate preparation, transparent reporting, protocol benchmarking where feasible, and complementary assessment matched to the scientific question.

Our findings show that most contemporary studies are limited to basic docking, whereas internal protocol verification and complementary quantitative assessment are used far less frequently. Improving reproducibility therefore depends less on developing new docking algorithms than on broader adoption of standardized protocols for structure preparation, internal model verification, and sequential validation of computational findings.

The validation workflow proposed here is a practical set of recommendations derived from current methodological approaches and the analysis of published studies. Its implementation should improve the reliability of molecular docking investigations of natural compounds and reduce false-positive conclusions.

## 10. Artificial Intelligence in Contemporary Molecular Docking

Over the past decade, artificial intelligence has become one of the most rapidly developing areas of computational chemistry and structural biology. Whereas machine-learning algorithms were initially used mainly to improve individual stages of virtual screening, contemporary AI systems increasingly encompass almost the entire computational workflow, from protein-structure prediction and generation of new chemical entities to automated interpretation of molecular docking results [12,13,118,119,120].

AI development is particularly relevant to natural compounds. Their pronounced structural complexity and conformational mobility, together with the limited number of experimentally characterized complexes, complicate the application of conventional molecular docking algorithms. Machine learning may improve virtual-screening efficiency but also raises new questions regarding prediction reliability, training-data quality, and reproducibility.

Artificial intelligence does not replace the fundamental physical principles of molecular interactions discussed in Section 7; rather, it provides a new level of structural-information processing. The main role of contemporary AI algorithms is not to replace classical docking, but to improve individual stages of the computational workflow and automate labor-intensive procedures for data preparation and analysis.

### 10.1. Artificial Intelligence in Structural Biology: New Opportunities and Current Limitations

The application of deep learning to three-dimensional protein-structure prediction is among the most important recent advances. AlphaFold2 enabled protein models to be generated with accuracy that, in many cases, approaches that of experimental structures, greatly expanding the number of potential molecular targets accessible to structure-based drug design [13]. The AlphaFold Protein Structure Database has made millions of predicted structures available for docking studies, including proteins for which no experimental data exist [16].

AlphaFold3 represents a further development, modeling not only individual protein structures but also complexes involving proteins, small molecules, nucleic acids, and metal ions [118]. This capability creates new opportunities for natural-product research, particularly for poorly characterized targets previously inaccessible to classical structure-based modeling.

Nevertheless, AI-predicted structures do not overcome the fundamental limitations of molecular docking. As discussed in Section 7.1, an accurate overall protein architecture does not guarantee correct local active-site geometry. Flexible loops, amino-acid side chains, and regions undergoing substantial ligand-induced conformational changes remain particularly challenging. These elements frequently determine specific hydrogen bonds and hydrophobic contacts that are critical to accurate docking of natural compounds.

Most AlphaFold models also represent only a single, most probable protein conformation. Many therapeutically important targets, including kinases, G-protein-coupled receptors, transporters, and large signaling proteins, function as ensembles of interconverting conformations. A single AI-predicted structure cannot fully capture the dynamic nature of binding and does not resolve the rigid-receptor limitation of conventional docking.

AI methods have thus considerably expanded the scope of structural biology and made thousands of new protein targets accessible. At present, however, they should be regarded primarily as a means of extending structural information rather than replacing experimental data or eliminating fundamental docking limitations. AI-predicted models require the same rigorous structure preparation and downstream validation as experimental PDB structures.

### 10.2. Artificial Intelligence in Molecular Docking: From Conventional Scoring Functions to Generative Models

Early molecular docking programs divided modeling into two separate stages: searching for ligand orientations and ranking the resulting poses using a predefined scoring function. Contemporary AI methods are gradually transforming both components by introducing new approaches to binding-energy estimation and direct prediction of protein-ligand complex structures.

Improvement of scoring functions remains the most widespread application of machine learning. Unlike conventional empirical and knowledge-based models, contemporary neural networks learn directly from large collections of experimental complexes and can identify complex nonlinear relationships governing complex formation. GNINA is a prominent example, using an ensemble of convolutional neural networks to rescore poses generated by conventional docking [12].

Such models often rank complexes more accurately than conventional scoring functions, particularly for chemical classes well represented in the training data. Their performance nevertheless depends strongly on training-set composition. Most contemporary neural networks were developed predominantly using synthetic drug-like molecules, whereas saponins, macrocycles, large polyphenols, highly glycosylated metabolites, and many other natural compounds are substantially underrepresented. The advantages of AI-based scoring for these compounds therefore remain to be established conclusively.

Generative models constitute the next development, with DiffDock being one of the best-known examples [14]. Rather than sequentially searching ligand orientations, DiffDock uses a deep-learning diffusion model to predict the most probable protein-ligand complex directly. This approach substantially reduces computational time and enables high-throughput virtual screening of very large compound libraries.

Generative models nevertheless retain the fundamental limitations of molecular docking. Their accuracy depends on representative training data, and independent studies indicate reduced performance for structurally atypical ligands and poorly characterized protein targets. Despite their considerable potential, these algorithms cannot yet be regarded as complete replacements for conventional docking and require the same downstream validation discussed in Section 9.

Artificial intelligence in contemporary docking software should be regarded primarily as a means of improving the efficiency of existing computational approaches rather than eliminating the fundamental limitations described in Section 7.

### 10.3. Generative Artificial Intelligence and Large Language Models in Natural-Product Research

In addition to advancing docking algorithms, AI is increasingly used in the design of new compounds and organization of computational research. Whereas neural-network scoring functions evaluate existing molecules, generative models can create new chemical structures with predefined properties, including predicted affinity for a selected protein target.

GENTRL is a prominent example that demonstrated automated design of novel protein inhibitors followed by experimental confirmation of activity [119]. In natural-product research, such approaches could produce optimized analogs of natural scaffolds that combine the structural diversity of natural metabolites with targeted improvement of drug-like properties.

Another rapidly developing direction is the use of large language models (LLMs) as an intelligent orchestration layer for computational workflows. LLMs do not calculate binding energies directly; instead, they can automate receptor and ligand preparation, parameter selection, the literature retrieval, report generation, and sequential execution of specialized software. ChemCrow, which combines a large language model with specialized chemical tools, illustrates this approach [120].

Such systems do not replace physical modeling of protein-ligand interactions; their main function is to automate the computational process and standardize individual research stages. The quality of the final results still depends on the methodological validity of the docking protocol and subsequent validation.

This is where AI may have its greatest impact on natural-product research. Automating structure preparation, verification of computational parameters, and protocol documentation can reduce the methodological errors identified in Section 8 and improve reproducibility.

### 10.4. Fully Automated Virtual Screening: New Opportunities and Current Limitations

Together, AI-based protein-structure prediction, neural-network scoring functions, generative models, automated structure preparation, and large language models are creating the basis for nearly fully automated virtual-screening platforms. Such systems integrate all major stages, from protein-target selection and receptor preparation to result analysis and report generation, within a single computational workflow.

This automation is particularly relevant to natural compounds. Contemporary databases contain hundreds of thousands of natural metabolites, and exhaustive analysis using conventional approaches requires substantial computational and human resources. Automated platforms can accelerate processing of these libraries, standardize individual procedures, and reduce subjectivity associated with manual structure preparation and parameter selection.

Automation alone, however, does not guarantee better results. If a computational protocol contains methodological errors, an automated platform merely reproduces them more rapidly and on a larger scale. Platform effectiveness is therefore determined by the validity of the computational protocol rather than by the degree of automation.

Widespread adoption of AI must consequently be accompanied by strict standardization of receptor and ligand preparation, mandatory internal protocol validation, and uniform criteria for result assessment. Otherwise, automation may increase false-positive findings rather than improve virtual-screening reliability.

### 10.5. Will Artificial Intelligence Transform Molecular Docking Methodology?

The rapid development of artificial intelligence naturally raises the question of whether it can fundamentally transform molecular docking methodology. The present analysis indicates that most current AI-based solutions improve individual stages of the existing computational workflow but do not eliminate the fundamental limitations discussed in Section 7.

Accurate protein-structure prediction does not resolve receptor conformational mobility. Neural-network scoring functions do not eliminate limitations arising from inadequate treatment of solvent, entropy, and specific intermolecular interactions. Generative models can accelerate candidate identification substantially, but their effectiveness remains dependent on training-data quality and does not remove the need for downstream validation.

Molecular docking is therefore likely to develop through integration of machine learning into a unified and reproducible computational workflow rather than replacement of classical algorithms by AI. Hybrid approaches combining conventional docking, molecular dynamics, binding-free-energy calculations, and intelligent automation of data preparation and analysis appear particularly promising.

The use of AI to standardize computational studies is especially important. Automated checks of structure preparation, modeling-parameter selection, computational-protocol validity, and documentation of every analytical stage could substantially improve reproducibility and reduce the methodological errors identified in Section 8.

The main contribution of artificial intelligence may lie less in creating new docking algorithms than in establishing more reliable, reproducible, and standardized computational workflows.

### 10.6. Section Summary

Artificial intelligence creates qualitatively new opportunities for natural-product research by expanding the range of accessible protein targets, accelerating virtual screening, and automating most stages of computational analysis. Contemporary AI technologies nevertheless do not eliminate the fundamental limitations of molecular docking associated with approximate descriptions of molecular interactions and the physical model of the protein-ligand complex.

Future progress will therefore be driven not by competition between conventional and AI-based methods but by their integration into unified computational protocols. Combining structural biology, machine learning, molecular docking, molecular dynamics, and fit-for-purpose multilevel validation represents a promising direction for natural-product research.

## 11. Conclusions

Molecular docking is among the most widely used tools in computer-aided drug discovery and occupies a central position in contemporary structure-based drug design. It is particularly valuable for natural compounds, which exhibit pronounced structural diversity, substantial stereochemical complexity, and a broad spectrum of potential biological targets. Specialized natural-product databases, high-quality protein structures, improved docking algorithms, and artificial intelligence have considerably expanded virtual-screening capabilities and accelerated the identification of new biologically active compounds.

The present analysis indicates, however, that future progress depends less on creating new algorithms than on improving the quality of their practical application. Despite major computational advances, most fundamental docking limitations persist. Restricted receptor flexibility, approximate treatment of solvent and entropy, sensitivity to protein and ligand preparation, and limitations of current scoring functions remain major sources of computational error and require cautious interpretation.

A central contribution of this review is the systematic methodological audit of contemporary natural-product docking studies. GPT-assisted assessment of 1127 publications, followed by full-text analysis of a random sample of 80 studies and independent human validation of 20 of those studies, provided a quantitative descriptive account of the reporting of key computational procedures. Stage 1/Stage 2 agreement was 98.3% across 525 paired decisions, while the human reviewer agreed with the Stage 2 classifications in 135 of 140 decisions (96.4%) within the 20-publication validation subsample. The five human-versus-Stage 2 discrepancies all arose when indeterminate AI labels (Unconfirmed or N/A) were resolved as No by the human reviewer, highlighting a specific classification-boundary issue rather than disagreement over clearly positive findings. Internal validation, including redocking and RMSD calculation, was reported in only a small proportion of studies, whereas procedures that do not directly establish docking-result reliability were substantially more frequent. These findings demonstrate a clear gap between current methodological recommendations and research practice. A further case-enriched assessment of 19 publications focused on redocking and redocking-RMSD classification. Two human reviewers agreed in all 38 decisions, whereas their consensus agreed with Stage 2 in 31/38 decisions (81.6%), showing that targeted review of rare and ambiguous cases can reveal classification errors that a small random validation sample may miss.

Methodological soundness cannot be inferred from the number of procedures reported. It depends on whether protein and ligand preparation, target selection, validation, and interpretation are appropriate to the study objective and claims. Molecular docking results should therefore be regarded not as definitive evidence of biological activity but as structural hypotheses requiring fit-for-purpose computational and experimental evaluation.

Based on a critical appraisal of the literature, this review proposes a practical protocol for molecular docking studies of natural compounds, including recommendations for structure preparation, internal protocol validation, assessment of complex stability, binding-free-energy estimation, and experimental confirmation. The proposed approach may support standardization and improve reproducibility irrespective of the docking software or natural-product class investigated.

Particular attention was paid to the influence of artificial intelligence on molecular docking. Contemporary AI technologies extend structural-biology capabilities, improve scoring functions, and automate individual stages of computational analysis, but they do not eliminate the fundamental limitations of conventional docking. The most promising direction is therefore not replacement of traditional computational methods by AI but their integration into reproducible workflows combining structural modeling, machine learning, molecular dynamics, binding-free-energy calculations, and experimental validation.

Future progress in natural-product research will depend not only on improved computational algorithms but, above all, on uniform methodological standards for conducting and evaluating studies. Greater reproducibility, fit-for-purpose multilevel validation, and closer integration of computational and experimental methods are essential for the effective use of molecular docking in natural-product drug discovery.

## Figures and Tables

**Table 1:** Comparison of widely used molecular docking software.

Software	Sampling Algorithm/Main Principle	Advantages	Limitations
AutoDock 4	Lamarckian genetic algorithm combined with an empirical free-energy function; partial flexibility of receptor side chains [10]	Free and open source; extensive validation literature; highly configurable	Slower than more recent alternatives; AutoDockTools requires user expertise
AutoDock Vina	Iterated local search using the Broyden-Fletcher-Goldfarb-Shanno algorithm and a simplified empirical scoring function [11]	Very fast; free; straightforward setup; a de facto standard for large-scale screening	Scoring is less accurate for absolute binding-energy estimates; limited treatment of specific interactions, including metals and aromatic stacking
Glide	Hierarchical systematic search employing sequential filters (HTVS, SP, and XP modes) [9]	Integrated commercial workflow with multiple screening modes and well-developed protein and ligand preparation tools	Commercial and costly; limited algorithmic transparency
MOE (Dock)	Combination of placement methods (Triangle Matcher and Alpha PMI) with multiple scoring functions (London dG and GBVI/WSA)	Integrated modeling environment encompassing preparation, docking, analysis, and QSAR; user-friendly graphical interface	Commercial; absence of a single peer-reviewed primary methodological publication complicates independent verification
GOLD	Genetic algorithm explicitly accounting for displacement of water molecules and partial receptor flexibility [8]	Extensively validated on diverse benchmarks; several alternative scoring functions within one package (GoldScore, ChemScore, and ASP)	Commercial; relatively slower than Vina and GNINA
LeDock	Heuristic simulated-annealing algorithm with a proprietary empirical scoring function	Free for academic use; compact workflow and efficient search implementation	No comprehensive peer-reviewed primary methodological publication; limited documentation and active support
FlexX	Incremental construction of the ligand fragment by fragment within the receptor active site [29]	One of the earliest industrial docking algorithms; rapid owing to incremental construction; incorporated into integrated platforms such as LeadIT and SYBYL	Commercial; performance is target-, ligand-set-, and benchmark-dependent
GNINA	Conventional sampling based on the AutoDock Vina/Smina engine followed by rescoring with an ensemble of convolutional neural networks [12]	Combines conventional sampling with convolutional-neural-network rescoring; GPU-enabled and open source	Efficient screening of large libraries requires a GPU; potential overfitting to unrepresentative training datasets
DiffDock	Generative diffusion model that predicts ligand poses end-to-end, without explicit conventional sampling and scoring [14]	Very rapid inference for large libraries; does not require a predefined active site as a rigid constraint	Sensitive to the distribution of PDB-derived training data; limited generalizability to atypical natural-product scaffolds; relatively new and less extensively validated on independent external datasets

**Table 2:** Operational definitions used in the methodological assessment.

Criterion	Operational Definition of a Positive Classification
Redocking	Explicit redocking of a crystallographic or reference ligand to test the docking protocol.
RMSD < 2 Å	A redocking RMSD below 2 Å; RMSD from an MD trajectory was not counted.
Known active ligand/reference comparator	A known active ligand, inhibitor, reference drug, or positive experimental comparator was used as a benchmark. This records the presence of a reference comparison; it does not by itself establish formal validation of pose reproduction, discrimination, ranking, or affinity prediction and was coded separately from native-ligand redocking.
MD simulation	A molecular-dynamics simulation of a protein-ligand system was reported.
MM/PBSA or MM/GBSA	A post-docking binding free-energy estimate using either method was reported.
Interaction analysis	Residue-level hydrogen-bond, hydrophobic, aromatic, or other non-covalent contacts were analyzed.
ADMET	Absorption, distribution, metabolism, excretion, toxicity, or an explicitly equivalent pharmacokinetic/toxicity assessment was reported.

**Table 3:** Frequency of methodological criteria in the Stage 1 corpus (ChatGPT Work, n = 1127; Supplementary Table S2) and the Stage 2 full-text sample (Claude Sonnet 5, n = 80; Supplementary Table S3).

Criterion	Corpus n/N	Corpus % (95% CI)	Sample n/N	Sample % (95% CI)
Redocking	27/1127	2.4 (1.7–3.5)	6/80	7.5 (3.5–15.4)
RMSD < 2 Å	19/1127	1.7 (1.1–2.6)	5/80	6.2 (2.7–13.8)
Known active/reference comparator	885/1127	78.5 (76.0–80.8)	70/80	87.5 (78.5–93.1)
MD simulation	444/1127	39.4 (36.6–42.3)	33/80	41.2 (31.1–52.2)
MM/PBSA or MM/GBSA	122/1127	10.8 (9.1–12.8)	10/80	12.5 (6.9–21.5)
Interaction analysis	1100/1127	97.6 (96.5–98.3)	79/80	98.8 (93.3–99.8)
ADMET	365/1127	32.4 (29.7–35.2)	28/80	35.0 (25.5–45.9)

**Table 4:** Cross-model agreement between Stage 1 (ChatGPT Work) coding and Stage 2 (Claude Sonnet 5) full-text verification in 75 directly paired publications.

Criterion	Agreement n/N	Agreement %	Discordant	Cohen’s Kappa
Redocking	73/75	97.3	2	0.786
RMSD < 2 Å	74/75	98.7	1	0.882
Known active/reference comparator	73/75	97.3	2	0.860
MD simulation	74/75	98.7	1	0.973
MM/PBSA or MM/GBSA	73/75	97.3	2	0.874
Interaction analysis	75/75	100.0	0	1.000
ADMET	74/75	98.7	1	0.970

**Table 5:** Minimum recommended protocol for molecular docking studies of natural compounds.

Stage	Status
Protein preparation	Required
Ligand preparation	Required
Selection of an experimental PDB structure	Preferred when an appropriate experimental structure is available; otherwise use a justified, validated predicted model
Molecular docking	Required
Redocking and RMSD assessment	Required when a suitable experimental reference complex is available; otherwise use an alternative benchmark. The RMSD definition and threshold must be prespecified and justified, with flexibility- and symmetry-aware interpretation for bulky natural products.
Known active ligand/reference comparator	Strongly recommended when a suitable known active/reference ligand is available; a comparator supports contextual benchmarking but is not, by itself, formal validation of docking-protocol performance
Interaction analysis	Required for structural interpretation, but not a substitute for protocol validation
ADMET analysis	Optional/complementary; relevant to developability rather than docking validation
Molecular dynamics	Recommended when dynamic stability or receptor flexibility is central to the question
MM/GBSA or MM/PBSA	Optional complementary relative energetic assessment; model-dependent
FEP	For lead optimization or rigorous relative comparisons of suitable congeneric series
Experimental confirmation	Required for strong claims of direct target engagement or novel biological activity

**Table 6:** Study objective and fit-for-purpose validation strategy.

Study Objective	Primary Validation Question	Most Appropriate Evidence	What the Evidence Does Not Establish
Reproduce a known pose	Can the protocol recover the experimental binding geometry?	Native-ligand redocking with pose RMSD; visual inspection of the reproduced interactions	Active–inactive discrimination, ranking accuracy, affinity, or biological activity
Virtual-screening enrichment	Can the method distinguish known actives from inactives or decoys?	Retrospective active/inactive or active/decoy benchmark; enrichment factor, ROC-AUC and, where relevant, early-recognition metrics	Quantitative binding affinity or direct target engagement
Rank a compound series	Does the predicted order follow experimental potency or affinity?	Prospective or external series with rank correlation and error analysis; preferably compounds not used to tune the protocol	Correct absolute affinity or efficacy in cells/organisms
Predict relative or absolute affinity	How closely do calculations reproduce measured binding energetics?	Comparison with experimental Kd, Ki, IC50 where mechanistically appropriate, or ΔG; model-appropriate statistics and uncertainty; FEP for suitable congeneric series	Biological activity, selectivity, exposure, or safety
Assess dynamic stability	Is the proposed complex stable within the simulated model and timescale?	Replicated MD with pose retention, RMSD/RMSF, interaction persistence, and convergence checks	Experimental pose correctness or binding affinity by itself
Interpret binding interactions	Are predicted contacts chemically and structurally plausible?	Residue-level interaction analysis, comparison with mutagenesis or known binding-site evidence	Formal validation of the docking protocol
Assess developability	Are pharmacokinetic or toxicological properties potentially acceptable?	ADMET prediction followed by relevant experimental assays	Docking accuracy, target engagement, or biological efficacy
Claim biological activity or target engagement	Does the compound act on the proposed target in an experimental system?	Biochemical/biophysical target-engagement assays, active and inactive controls, followed by cellular or in vivo confirmation as appropriate	Mechanistic causality beyond the scope of the experiments performed

## Data Availability

The original contributions presented in this study are included in the article and Supplementary Materials. Further inquiries can be directed to the corresponding author.

## Supplementary Materials

The following supporting information can be downloaded at: https://www.mdpi.com/article/10.3390/molecules31183228/s1, Supplementary Methods S1: reproducible AI-assisted classification prompt, input structure, label rules, ambiguity handling, and model/version information; the complete Supplementary Methods S1 file accompanies the revision and contains the verbatim user-level prompts and workflow specification. Proprietary platform-level system prompts were not accessible to the authors. Supplementary Table S1: Recommended protein targets and reference PDB structures for molecular docking studies of natural products, grouped by therapeutic area (corresponds to Section 6 of the main text). Supplementary Table S2: Study-level characteristics and validation features assessed during evidence synthesis. Supplementary Table S3: Full-text methodological assessment of 80 publications randomly sampled from the 1127-publication corpus. Supplementary Table S4: Human validation sample (n = 20). Supplementary Table S5: Targeted case-enriched human validation of AI-assisted redocking and redocking-RMSD classification.
