# Supplementary material for: Molecular Docking of Natural Products: Critical Appraisal of Current Methodology and Practical Guidelines
Source: Molecules. 2026 Sep 12;31(18):3228. doi: 10.3390/molecules31183228 (PMC13609022; doi:10.3390/molecules31183228)
Supplement: Supplementary file 1 [file molecules-31-03228-s001.zip › Supplementary_Methods_S1_AI_workflow.pdf]

Supplementary Methods S1

Reproducible AI-assisted classification workflow for the methodological audit

This supplement documents the user-controlled components of the AI-assisted evidence-synthesis workflow: model environment, article input structure, instruction templates, output schema, classification rules, ambiguity handling, and human verification. It is intended to permit reapplication of the same coding logic to the archived article records.

S1. Scope of prompt disclosure

The verbatim user-level instruction templates are reproduced below. ChatGPT Work and Claude are commercial hosted services whose platform-level system prompts and internal orchestration messages were not displayed to or controlled by the authors. Those hidden messages cannot be reproduced and were not reconstructed. Reproducibility therefore refers to the disclosed user-level protocol, model identification, structured input, decision rules, and archived article-level outputs.

S2. Models and roles

Stage 1: ChatGPT Work (OpenAI), powered by GPT-5.6 Sol; structured coding of the 1,127-record corpus.

Stage 2: Claude Sonnet 5 (Anthropic); detailed assessment of the random 80-publication sample using available full texts.

Human verification: one reviewer assessed the random 20-publication seven-criterion subset; two independent reviewers assessed the targeted 19-publication redocking/RMSD set, followed by consensus recording.

S3. Article input structure

Each record was supplied individually in the following fixed order:

ARTICLE TITLE: [verbatim title]
DOI/IDENTIFIER: [DOI or bibliographic identifier]
SOURCE TYPE: [full text / accessible article text / abstract-indexed text]
ARTICLE TEXT: [retrieved text supplied for classification]
END OF ARTICLE RECORD

The model was instructed to use only the supplied record and not to infer an unreported procedure. The source-type field was retained so that incomplete access could be represented as Unconfirmed rather than silently treated as No.

S4. Verbatim Stage 1 user-level instruction template

Using only the supplied article record, classify each of the seven prespecified criteria as Yes, No, Unconfirmed, or N/A. For every criterion, return the label and concise supporting evidence or the location of that evidence. Do not infer that a method was performed when it is not explicitly reported. Do not count RMSD from a molecular-dynamics trajectory as redocking RMSD. Do not treat docking of a known active ligand/reference comparator as native-ligand redocking unless the crystallographic/reference pose is explicitly redocked to test pose reproduction. Use the operational definitions and output format supplied below.

S5. Verbatim Stage 2 user-level instruction template

Assess the supplied article text independently using the same seven operational definitions. Classify every criterion as Yes, No, Unconfirmed, or N/A and cite concise article-level evidence for the decision. Use Yes only when the criterion is explicitly documented. Use No when the supplied full text is sufficient for assessment and the procedure is not reported. Use Unconfirmed when access or wording is insufficient for a defensible binary decision. Use N/A only when the criterion is logically non-applicable. Distinguish redocking-pose RMSD from molecular-dynamics trajectory RMSD, and distinguish a known active/reference comparator from native-ligand redocking. Do not consult the Stage 1 classification when making the initial Stage 2 judgment.

S6. Operational definitions

**Criterion**	**Operational definition**
Redocking	Explicit redocking of a crystallographic or reference ligand to test the docking protocol.
Redocking RMSD <2 Å	A pose RMSD below 2 Å obtained by comparing the redocked ligand with its crystallographic/reference pose; MD-trajectory RMSD was excluded.
Known active ligand/reference comparator	A known active ligand, inhibitor, reference drug, or experimental comparator used as a benchmark; this was recorded separately from native-ligand redocking and was not treated as formal pose-validation evidence.
MD simulation	A molecular-dynamics simulation of a protein–ligand system was reported.
MM/PBSA or MM/GBSA	A post-docking binding-free-energy estimate using either method was reported.
Interaction analysis	Residue-level hydrogen-bond, hydrophobic, aromatic, or other non-covalent contacts were analysed.
ADMET	Absorption, distribution, metabolism, excretion, toxicity, or an explicitly equivalent pharmacokinetic/toxicity assessment was reported.

S7. Label rules

Yes: Explicit evidence meets the operational definition.

No: The assessable record is sufficiently complete and the procedure is not reported or the reported procedure clearly does not meet the definition.

Unconfirmed: Incomplete access, ambiguous wording, conflicting evidence, or insufficient detail prevents a defensible Yes/No decision.

N/A: The criterion is logically non-applicable; for example, a qualifying redocking RMSD cannot be assessed when no redocking was performed.

S8. Required output schema

Article identifier | Criterion | Classification (Yes/No/Unconfirmed/N/A) | Supporting evidence | Evidence location | Access limitation/ambiguity note

S9. Ambiguity and quality-control workflow

Preserve Unconfirmed or N/A rather than automatically mapping either label to No.

Check every RMSD-positive decision to establish whether it refers to redocking pose reproduction or to an MD trajectory.

Check every redocking-positive decision to establish whether a crystallographic/reference ligand was actually redocked; docking a comparator or visually aligning poses alone is insufficient.

Retain concise evidence and an article location for auditability.

Compare Stage 1 and Stage 2 only after the independent Stage 2 classification is recorded.

Refer ambiguous or discordant cases to human review; preserve the original AI label and the adjudicated consensus as separate fields.

S10. Article-level audit trail

Individual Stage 1 and Stage 2 classifications are reported in Supplementary Tables S2 and S3. The random 20-publication human-validation comparison is reported in Supplementary Table S4. The targeted two-reviewer redocking/RMSD validation, consensus decisions, and supporting evidence are reported in Supplementary Table S5. Together, these files preserve the model outputs, human decisions, disagreement direction, and evidence required to audit the reported agreement calculations.
