# Supplementary material for: Molecular Docking of Natural Products: Critical Appraisal of Current Methodology and Practical Guidelines
Source: Molecules. 2026 Sep 12;31(18):3228. doi: 10.3390/molecules31183228 (PMC13609022; doi:10.3390/molecules31183228)
Supplement: Supplementary file 1 [file molecules-31-03228-s001.zip › Supplementary_Tables_S1.pdf]

Supplementary Materials

Supplementary Table S1. Recommended protein targets and reference PDB structures for molecular docking studies of natural products, grouped by therapeutic area (corresponds to Section 6 of the main text).

Note: reference numbers in square brackets correspond to the continuous bibliography numbering of the main manuscript. Where the same PDB entry is cited under two therapeutic areas (e.g., SARS-CoV-2 Mpro under §6.3 and §6.12; EGFR under §6.1 and §6.9), this reflects the target's relevance to more than one section and is not a duplication error.

Category	Target	Recommended PDB	Reference
6.1 Oncology	Epidermal Growth Factor Receptor (EGFR)	1M14	[42]
	Topoisomerase IIα	4FM9	[43]
	Tubulin (αβ-tubulin)	4O2B	[44]
	Cyclin-Dependent Kinase 2 (CDK2)	1HCK	[45]
	p53/MDM2	1YCR	[46]
6.2 Infectious diseases (bacterial)	DNA gyrase (GyrB)	4URM	[47]
	Penicillin-Binding Protein 2a (PBP2a)	3ZG5	[48]
	Dihydrofolate reductase (DHFR)	1U72	[49]
6.3 Viral infections	Main protease (Mpro/3CLpro), SARS-CoV-2	6LU7	[50]
	HIV-1 protease	1HVR	[51]
	HIV-1 reverse transcriptase	4PWD	[52]
	Influenza neuraminidase	2HU4	[53]
6.4 Inflammation	Cyclooxygenase-2 (COX-2)	5IKR	[54]
	Tumor Necrosis Factor-α (TNF-α)	2AZ5	[55]
	NF-κB (p50/p65–IκBα complex)	1NFI	[56]
	5-Lipoxygenase (5-LOX)	3V99	[57]
6.5 Neurology	Acetylcholinesterase (AChE)	2ACE (native TcAChE)	[58]
	Butyrylcholinesterase (BuChE)	4BDS	[59]
	β-Secretase 1 (BACE1)	2ZHV	[60]
	Monoamine Oxidase A (MAO-A)	2Z5X	[61]
	Monoamine Oxidase B (MAO-B)	2V5Z	[62]
6.6 Diabetes mellitus	α-Glucosidase	3A4A	[63]
	Human pancreatic α-amylase	2CPU	[64]
	Dipeptidyl Peptidase-4 (DPP-4)	2ONC	[65]
	PPAR-γ	2PRG	[66]
	Protein Tyrosine Phosphatase 1B (PTP1B)	1PTY	[67]
6.7 Cardiovascular diseases	Angiotensin-Converting Enzyme (ACE)	1O86	[68]
	HMG-CoA reductase	1HWK	[69]

Category	Target	Recommended PDB	Reference
	Thrombin	1HRT	[70]
	Coagulation Factor Xa	2P16	[71]
	Phosphodiesterase-5 (PDE5)	1UDT	[72]
6.8 Contemporary targets: inflammation, immunity, oxidative stress	NLRP3 inflammasome	7ALV	[73]
	IKKβ (IKBKB)	4KIK	[74]
	mPGES-1	4YL3	[75]
	iNOS (NOS2)	3E7G	[76]
	TLR4–MD2	3FXI	[77]
	Keap1 (Kelch domain)	4IQK	[78]
6.9 Contemporary anticancer targets: PI3K/AKT/mTOR pathway and apoptosis	PI3Kα	6OAC	[79]
	AKT1	3O96	[80]
	mTOR kinase	4JSV	[81]
	EGFR (alternative structure)	1M17	[42]
	VEGFR2 (KDR)	3VO3	[82]
	BCL-2	4LVT	[83]
6.10 Contemporary metabolic targets (obesity, diabetes, metabolic syndrome)	AMPK	4CFF	[84]
	SGLT2	7VSI	[85]
	GLP-1R	7S15	[86]
	Aldose reductase (AKR1B1)	1ADS	[87]
	Fatty acid synthase (FASN)	2PX6	[88]
	Acetyl-CoA carboxylase (ACC1)	3JRW	[89]
6.11 Contemporary antibacterial targets: cell wall and bacterial division	MurA	1UAE	[90]
	MurB	1MBT	[91]
	FabI	1C14	[92]
	FtsZ	1RQ7	[93]
	Topoisomerase IV (ParE/ParC)	3RAE	[94]
	LpxC	2VES	[95]
6.12 Contemporary antiviral targets	SARS-CoV-2 Mpro (3CLpro)	6LU7	[50]
	SARS-CoV-2 PLpro	6WZU	[96]
	SARS-CoV-2 RdRp (NSP12)	7BV2	[97]
	SARS-CoV-2 helicase (NSP13)	6XEZ	[98]

Category	Target	Recommended PDB	Reference
	SARS-CoV-2 NSP16/NSP10 (2′-O-methyltransferase)	6WKS	[99]
	HCV NS3/4A protease	3KEE	[100]
	HCV NS5B RNA polymerase	2XI3	[101]
	RSV F protein	5TPN	[102]
6.13 Contemporary targets in neurodegenerative diseases	GSK-3β	1Q5K	[103]
	LRRK2	7LI4	[104]
	COMT	3BWM	[105]
	Adenosine A2A receptor (A2AR)	3EML	[106]
	HDAC6	5EDU	[107]
	Tau protein (filaments)	5O3L	[108]
	TrkB	4ASZ	[109]
	SIRT1	4I5I	[110]
